# Charting the evolution of approaches employed by the Global Alliance for Vaccines and Immunizations (GAVI) to address inequities in access to immunization: a systematic qualitative review of GAVI policies, strategies and resource allocation mechanisms through an equity lens (1999–2014)

**DOI:** 10.1186/s12889-015-2521-8

**Published:** 2015-11-30

**Authors:** Gian Gandhi

**Affiliations:** grid.420318.c000000040402478XUnited Nations Children’s Fund, New York, USA

**Keywords:** Global alliance for vaccines and immunization, GAVI alliance, Gavi—the vaccine alliance, Equity of access to vaccines, Equity of access to immunization, Immunization coverage disparities, Gender inequality in immunization, Immunization resource allocation mechanisms, GAVI budget caps, GAVI eligibility, GAVI policies.

## Abstract

**Background:**

GAVI’s focus on reducing inequities in access to vaccines, immunization, and GAVI funds, − both between and within countries - has changed over time. This paper charts that evolution.

**Methods:**

A systematic qualitative review was conducted by searching PubMed, Google Scholar and direct review of available GAVI Board papers, policies, and program guidelines. Documents were included if they described or evaluated GAVI policies, strategies, or programs and discussed equity of access to vaccines, utilization of immunization services, or GAVI funds in countries currently or previously eligible for GAVI support. Findings were grouped thematically, categorized into time periods covering GAVI’s phases of operations, and assessed depending on whether the approaches mediated equity of opportunity or equity of outcomes between or within countries.

**Results:**

Serches yielded 2816 documents for assessment. After pre-screening and removal of duplicates, 552 documents underwent detailed evaluation and pertinent information was extracted from 188 unique documents. As a global funding mechanism, GAVI responded rationally to a semi-fixed funding constraint by focusing on between-country equity in allocation of resources. GAVI’s predominant focus and documented successes have been in addressing between-country inequities in access to vaccines comparing lower income (GAVI-eligible) countries with higher income (ineligible) countries. GAVI has had mixed results at addressing between-country inequities in utilization of immunization services, and has only more recently put greater emphasis and resources towards addressing within-country inequities in utilization to immunization services. Over time, GAVI has progressively added vaccines to its portfolio. This expansion should have addressed inter-country, inter-regional, inter-generational and gender inequities in disease burden, however, evidence is scant with respect to final outcomes.

**Conclusion:**

In its next phase of operations, the Alliance can continue to demonstrate its strength as a highly effective multi-partner enterprise, capable of learning and innovating in a world that has changed much since its inception. By building on its successes, developing more coherent and consistent approaches to address inequities between and within countries and by monitoring progress and outcomes, GAVI is well-positioned to bring the benefits of vaccination to previously unreached and underserved communities towards provision of universal health coverage.

**Electronic supplementary material:**

The online version of this article (doi:10.1186/s12889-015-2521-8) contains supplementary material, which is available to authorized users.

## Background

Gavi, the Vaccine Alliance (formerly known as the ‘GAVI Alliance’, and prior to that the ‘Global Alliance for Vaccines and Immunization’ and hereafter referred to as ‘GAVI’, ‘the Alliance’ or ‘the GAVI Alliance’) was launched in the year 2000. After fifteen years of operations, GAVI has developed a strategic framework to articulate priorities and program investments for its next five-years of operations (2016 through 2020). The GAVI Board - which governs activities funded by the Alliance - requested that in its next phase of operations, GAVI deepen its focus on improving the coverage and equitable distribution of immunization services in GAVI countries^1^ [1–3].

GAVI’s new 2016–2020 strategy and operational plans will ultimately be framed within the broader context of the Post-2015 sustainable development agenda. Recent consultations on the Post-2015 agenda for health suggest the need for equity in access and utilization of healthcare between and within countries. As such, people (rather than states or institutions) will be at the heart of the sustainable development agenda [4–9]. This builds on the growing recognition that existing global development goals - with their focus on national averages - have resulted in unequally distributed gains, and at worst have inadvertently exacerbated inequities in access within countries, particularly for child health [10–14]. By contrast, a growing body of evidence suggests that addressing these inequities could drastically reduce child mortality in low and middle income countries [15, 16].

GAVI’s approach in 2016 and beyond will need to draw upon the Alliance’s previous successes as well as areas identified for improvement. Many of these areas have been documented in a cadre of retrospective evaluations since GAVI’s formation and over its previous two phases of operations [Formation and Phase I (1999–2005), and Phase II (2006–2010)] [17–26]. GAVI’s operations in its next strategic period will also draw upon more recent results which are being gleaned in ongoing retrospective [27]^2^ and prospective evaluations [28] for the current phase of GAVI’s operations, Phase III (2011–2015).

Despite a wealth of information, GAVI’s efforts to address inequities in immunization —through its strategies, policies and resource allocation mechanics—have not been explicitly mapped out.^3^ This paper delineates how GAVI’s focus on equity has evolved and seeks to identify the range of opportunities that may further ameliorate disparities in access and effective utilization of immunization services.

## Methods

### Scope and objectives

A systematic qualitative review of the literature^4^ was undertaken to assess how GAVI’s approach to address *equity/inequity*^5^*in immunization* has evolved over time. Since equity is itself a comparative concept, this paper focuses on various comparisons between countries, and within countries.

- *Between-country equity* is concerned with differences between countries, either comparing GAVI-eligible and ineligible countries, or comparing GAVI-eligible countries to one another;
- *Within-country equity* is concerned with differences amongst populations residing within the national borders of a GAVI-eligible country, where populations may be stratified either by geography, socioeconomic status, or gender.

This paper separates out the ‘differences’ in which GAVI’s strategic, policy and programmatic choices have addressed or have been driven by concerns related to *equity of opportunity* as opposed to *equity of outcomes* [29, 30]. This paper considers equity of opportunity in two interlinked ways:

(i) Opportunity to access specific antigens/ vaccines
(ii) Opportunity to access (or be allocated) GAVI resources

This paper considers equity of outcomes in terms of the following intermediate and final outcomes:

(iii). Intermediate outcomes in terms of coverage or utilization of immunization services
(iv). Final outcomes in terms of impact on vaccine preventable disease (VPDs)

Not all combinations of between/within country equity, equity of opportunity/outcomes are considered to be relevant for this analysis . For example, since GAVI has provided support to national rather than subnational governments, it is only relevant to assess GAVI’s impact on the equity of opportunity to access GAVI resources between countries (rather than within countries). By extension, since GAVI helps facilitate vaccine introductions into National Immunization Programs (NIPs), and since NIPs mostly aim to deliver a single schedule of vaccines across the country,^6^ it is most relevant to assess GAVI’s contribution to equity of opportunity to access specific antigens/ vaccines between countries (rather than within countries).

Finally, when assessing strategies, policies or programs that concern the distribution of- GAVI resources, this paper makes distinctions between whether GAVI’s distributive approaches have been driven by attempts to ensure *horizontal equity* (i.e. ‘equal treatment for equivalent needs’) or *vertical equity* (‘preferential treatment for those with greater health needs’) [31, 32].

### Literature review strategy

Table 1 summarizes the literature review strategy. Conventional steps of a literature review were followed, i.e. searching the literature, extracting relevant information, and assessing the quality of included papers. Two open-source electronic databases (National Library of Medicine’s PubMed, Google Scholar) were included in the search. Given the importance of specific grey literature to this review, one website (the GAVI Alliance website) and a private electronic archive (belonging to United Nations Children’s Fund (UNICEF)) were searched. The electronic archive was searched because from 1999 until 2008, UNICEF housed the GAVI Secretariat and hence held a small electronic repository of GAVI documents; furthermore the GAVI Secretariat published its Board papers only from the moment it gained independence from UNICEF; i.e. from 2009 onwards.

**Table 1:** Literature review strategy

Electronic database	PubMed, Google Scholar
Websites searched	GAVI Alliance website
Electronic archives	UNICEF
Additional sources	Supporting references of all papers meeting criteria (below) yielded from searching the sources (above) were also reviewed.
Inclusion criteria	• Dealt explicitly with the GAVI Alliance (e.g. described findings from GAVI evaluations; detailed GAVI programs, policies, and/or resource allocation decisions) • Explicitly discussed inequities/inequalities in access to vaccines, inequities/inequalities in coverage of immunization services, or inequities/inequalities in access/allocation to GAVI funds • Related to one or more country which is, or has been, eligible to receive GAVI support since GAVI’s inception
Search updated to	01 November 2014
Restriction on language	English only
Restriction on year of publication	Published between 1998 and 2014 inclusive
Search terms	Terms used for PubMed searches were “Global Alliance for Vaccines and Immunizations” OR “GAVI Alliance” OR “(GAVI)” AND “equity” OR “inequity” OR “equitable” OR “inequitable” OR “disparity”, OR “inequality”, OR “access to funds”, OR “access to vaccines”, OR “resource allocation”, OR “coverage deficits”, OR “immunization coverage”.
	For Google Scholar, many of the same search terms were used; notably “GAVI Alliance” AND “equity” OR “equality” OR “access” OR “coverage” OR “resource allocation”
	In reviewing unpublished grey literature and GAVI documents, key word searches were performed using the same terms as used for Google Scholar searches.
Categorization of papers identified	Papers meeting the inclusion criteria were categorized according to three time periods (Phase I: 1999–2005; Phase II: 2006–2010; Phase III: 2011–2015) and according to GAVI’s main policies, strategies, programs (strategy and work plan, vaccine priorities and new vaccine support, country eligibility policy, large countries and budget caps policies, program filters, cash-based support for program/system strengthening, vaccine introduction grants, financial sustainability policy, supply and procurement strategy, fragile states policy, program funding prioritization, gender policy)

Grey literature focused on papers written/published by the GAVI Secretariat (i.e. GAVI Board papers, GAVI Committee^7^ papers, GAVI policies/strategies/program documents) or independent evaluations of GAVI policies/programs. Hereafter, these sources are referred to as ‘GAVI documents’.

The pool of evidence was restricted by year of publication in order to identify papers dealing with the period immediately before GAVI’s inception to ascertain the motivations for the formation of GAVI, as well as the subsequent period that the GAVI Alliance has functioned. Inclusion criteria and search terms were selected in order to focus the review to the scope and objectives outlined above.

Information was extracted from all identified documents meeting inclusion criteria and based on an assessment of relevance to the study objectives and scope. Extracted information from all papers included in the review were categorized thematically according to eleven strategy, policy, and programmatic pillars that have defined GAVI since inception:

1. Overarching strategy and workplans
2. Vaccine priorities and New Vaccine Support (NVS)
3. Supply and procurement strategies
4. Country eligibility policies and program filters
5. Large countries and budget cap policies
6. Cash-based support for program/system strengthening and guidelines
7. Vaccine introduction grants (VIGs), policies and guidelines
8. Financial sustainability (co-financing) policies
9. ‘Fragile States’ policies
10. Gender policy
11. Vaccine and cash-based program funding prioritization

GAVI itself, its policies, and its strategies have changed over time. The assessment therefore applies a temporal lens to further collate the information. Each of the above-mentioned thematic categorizations have been sub-divided according to three time periods associated with GAVI’s operational ‘phases’:

I. Formation/Phase I (1999–2005)—The formative years and GAVI’s first phase of operations
II. Phase II (2006–2010)—The second phase of GAVI Alliance operations
III. Phase III (2011–2015)—The third and current phase of GAVI Alliance operations

Finally, despite the non-clinical nature of the subject matter, the PRISMA guidelines [33] were followed as appropriate to conduct the literature review. The PRISMA Checklist has been completed and is provided in Additional file 1.

### Handling of bias and validity

The methods for this review of qualitative evidence draw upon guidance delineated by Booth (2001) and Shaw et al. (2004) concerning the conduct of systematic review and literature search of qualitative evidence [34, 35]. Accordingly, methods employed for source identification have been designed to a) identify major “schools of thought”; b) bring different views through multi- disciplinary searches; c) draw upon complementary electronic and manual search techniques to ensure that materials are not missed. Rather than rejecting sources a priori based on the study design or where the information was published, instead additional weight has been assigned to the findings or narrative explanations from more robust and/or unbiased evidence in summarizing the evidence overall. These approaches lend themselves to greater heterogeneity of inputs which was deemed permissible in the context of this review since much of the material reviewed is neither primary nor secondary research (but rather policies, program guidelines, meeting minutes etc.). This review was approached with cognizance of the criteria to critically appraise findings from qualitative research [36]. Issues of error, bias and validity as well as trustworthiness of the sources themselves were carefully considered [37, 38].

Given the significant use of grey literature, there are a number of ways in which bias, validity and trustworthiness have been handled. Regarding validity and trustworthiness, the focus of grey literature searches has been on ‘GAVI documents’ (GAVI Board and Committee Papers and Minutes, formally commissioned rigorous independent evaluations, GAVI program guidelines, GAVI progress reports etc.). In general, GAVI Board/Committee Papers and GAVI Board/Committee Minutes are reviewed and scrutinized by many stakeholder organizations and constituencies that make up the Alliance including normative agencies such the WHO, multilateral agencies such as the World Bank, and experts from sovereign governments (both within Ministries of Health within GAVI countries, and Ministries of Foreign Affairs/Ministries of Development Cooperation in donor governments). GAVI Board Minutes are also subject to a formalized sign-off process to ensure that all parties are satisfied that the Minutes are a true reflection of proceedings. As such, GAVI documents - and particularly the Minutes and Board-approved policies and programs cited - largely reflect the consensus of the Alliance, rather than the biased position of any single actor. Most importantly, to minimize some reporting biases (e.g. associated with the groupthink of the Alliance), aside from the peer-reviewed citations, maximum weight was given to the independent evaluations of GAVI programs and policies.

Some reporting biases could not be controlled such as publication bias (e.g. associated with the publication and reporting of positive results). To address these deficiencies, absence of specific and relevant evidence is highlighted (e.g. where GAVI work plans note a particular intervention to address inequities, but no results have been reported/published).

## Results

**Fig. 1:**
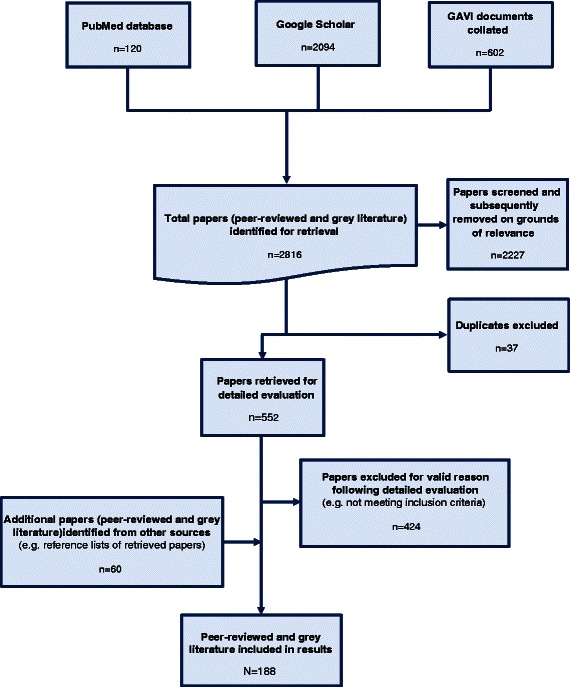
Schematic of article selection

**Fig. 2:**
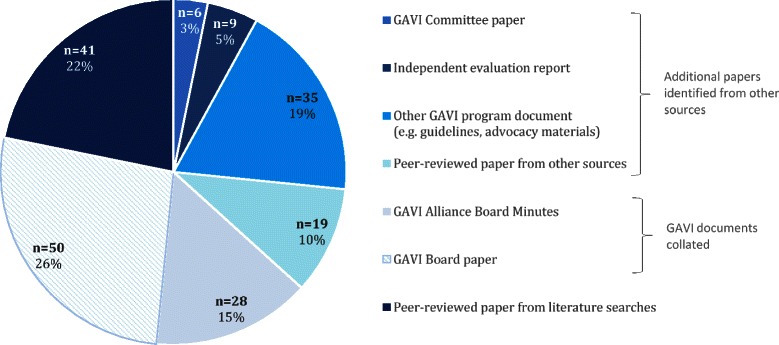
Types of sources of information (*N* = 188)

PubMed and Google Scholar searches yielded 120 and 2094 results respectively, while 602 GAVI Board papers were collated from online and electronic archives. Following a review of titles and abstracts, 2227 papers were excluded due to lack of relevance. After removal of duplicates, a total of 544 unique documents underwent detailed evaluation. Based on these reviews 424 papers were excluded because they were found not to meet the inclusion/exclusion criteria, but a further 60 papers were identified (e.g. from reference lists). Pertinent information was extracted from the remaining 188 unique documents. Fig. 1 illustrates the article selection process while Fig. 2 details the composition of documents included in the final results. (See Additional file 2 for further details.)

Table 2 summarizes the main results from the qualitative systematic review. It illustrates each of the eleven strategy/policy/program pillars across the three phases of GAVI operations for equity of outcomes and equity of opportunity (final/intermediate). The table categorizes the findings according to:

- Whether a conceptual approach was articulated to address between- or within- country inequities
- The strength/breadth of the evidence to illustrate the effect/impact of the GAVI strategy, policy, or program in question
- Whether this evidence suggests positive and/or negative impacts on the particular inequities
- Whether it is not relevant to assess (because there is no way that a strategy, policy, or program here could directly mediate equity impact)
- Whether it is not applicable (because there was no specific strategy, policy, or program articulated for particular phase of operations)

**Table 2:** Summary of main findings

Key GAVI strategy, policy and programme pillar	EQUITY OF OPPORTUNITY				EQUITY OF OUTCOME (Intermediate)					EQUITY OF OUTCOME (Final)	
	Between-country equity comparing				Between-country equity comparing		Within-country equity comparing			Between-country equity comparing	
	(i) access to new/underutilized vaccines:		(ii) access to GAVI resources:		(iii) utilization of immunization services:		(iii) utilization of immunization services			(iv) VPD burden among:	
GAVI phase of operations	GAVI and non-GAVI countries	Individual GAVI countries	GAVI and non-GAVI countries	Individual GAVI countries	GAVI and non-GAVI countries	Individual GAVI countries	Geography (between district)	Socio-economic status	Gender	GAVI and non-GAVI countries	Individual GAVI countries
1. Overarching strategy and workplans											
Phase I	++	-(+)	++	-(+)	+(+)	+(+)	++	-(+)	++	+	+
Phase II	++	-(+)	++	-(+)	+(+)	+(+)	++	-(+)	--	-(+)	-(+)
Phase III	+	-(+)	+	-(+)	+	+	+	+	+	--	--
2. Vaccine Priorities, NVS and guidelines											
Phase I	++	-(+)	++	-(+)	N/R	N/R	+	--	+	++	-(+)
Phase II	++	++	++	++	N/R	N/R	--	--	--	++	++
Phase III	+	+	+	+	N/R	N/R	+	+	+	+	+
3. Supply and procurement strategies											
Phase I	+(+)	N/R	+(+)	N/R	N/R	N/R	N/R	N/R	N/R	N/R	N/R
Phase II	+(+)	N/R	+(+)	N/R	N/R	N/R	N/R	N/R	N/R	N/R	N/R
Phase III	+	N/R	+	N/R	N/R	N/R	N/R	N/R	N/R	N/R	N/R
4. Country eligibility and program filters											
Phase I	+	+(+)	+	+(+)	+(+)	+(+)	--	--	--	--	--
Phase II	+(+)	+(+)	+(+)	+(+)	+(+)	+(+)	--	--	--	--	--
Phase III	+(+)	+(+)	+(+)	+(+)	+(+)	+(+)	--	--	--	--	--
5. Large countries and budget cap policies											
Phase I	N/R	N/R	N/R	N/R	--	--	-(+)	--	--	--	--
Phase II	N/R	N/R	N/R	N/R	--	--	-(+)	--	--	--	--
Phase III	N/R + N/A	N/R + N/A	N/R + N/A	N/R + N/A	N/R + N/A	N/A	N/A	N/A	N/A	N/A	N/A
6. Cash-based support and guidelines											
Phase I	N/R	N/A	N/R	N/A	--	--	-(+)	--	--	--	--
Phase II	N/R	N/A	N/R	N/A	--	--	+(+)	+(+)	--	--	--
Phase III	N/R	N/A	N/R	N/A	--	--	+	+	+	--	--
7. Vaccine introduction grants (VIGs) policies											
Phase I	N/R	N/A	N/R	N/A	--	--	--	--	--	--	--
Phase II	N/R	N/A	N/R	N/A	--	--	--	--	--	--	--
Phase III	N/R	N/A	N/R	N/A	--	--	--	--	--	--	--
8. Financial sustainability (Co-financing)											
Phase I	N/R	--	N/R	--	N/R	N/R	--	--	--	--	--
Phase II	N/R	+	N/R	+	N/R	N/R	--	--	--	--	--
Phase III	N/R	+	N/R	+	N/R	N/R	--	--	--	--	--
9. ‘Fragile States’ policies											
Phase I	--	-(+)	--	-(+)	--	--	--	--	--	--	--
Phase II	--	--	--	--	--	--	+	--	--	--	--
Phase III	+	+	+	+	+	+	+	+	+	+	--
10. Gender Policy											
Phase I	N/R + N/A	N/A	N/R + N/A	N/A	N/R + N/A	N/A	N/A	N/A	N/A	N/R + N/A	N/A
Phase II	N/R	N/R	N/R	N/R	--	--	N/R	N/R	+	--	--
Phase III	N/R	N/R	N/R	N/R	--	--	N/R	N/R	+	--	--
11. Programme prioritization											
Phase I	N/R + N/A	N/A	N/R + N/A	N/A	N/R + N/A	N/A	N/A	N/A	N/A	N/R + N/A	N/A
Phase II	N/R + N/A	N/A	N/R + N/A	N/A	N/R + N/A	N/A	N/A	N/A	N/A	N/R + N/A	N/A
Phase III	N/R	--	N/R	--	N/R	--	--	--	--	N/R	--

Key:

^+^Conceptual approach articulated that should in theory address inequities but no supporting evidence to assess effect/impact on equity

^+(+)^Conceptual approach plus limited evidence (in terms of breadth and/or robustness) to suggest positive and/or negative impacts on equity

^++^Conceptual approach plus limited evidence (in terms of breadth and/or robustness) to suggest positive impact on equity\

^+++^Conceptual approach plus significant evidence (in terms of breadth and/or robustness) to demonstrate positive impact on equity

^-(+)^No conceptual approach mentioned but limited evidence (in terms of breadth and/or robustness) of positive and/or negative impacts on equity

^−−^No conceptual approach mentioned nor any evidence to illustrate impact on equity

^N/R^Not relevant (i.e. Strategy/policy/programme cannot directly mediate equity impact)

^N/A^Not applicable (i.e. No specific strategy/policy/programme articulated for particular phase of operations)

### Overarching strategy and workplans

#### Formation/Phase I

Evidence from the formative years of GAVI, and its precursor, the Children’s Vaccine Initiative (CVI), suggests that one of the key motivating factors for setting up a partnership to focus on vaccines and immunizations was to address the disparities in access to so-called ‘new and under-utilized vaccines’ *between* high income and lower income countries, as observed by GAVI’s founders [39–41]. GAVI’s founders included philanthropic institutions (Bill & Melinda Gates Foundation and the Rockefeller Foundation), a small number of sovereign donors (the UK, USA, Norway, Netherlands, Denmark, and Sweden) and partner multilateral agencies (UNICEF, WHO and the World Bank) [17, 40].Individuals from these institutions coalesced around the notion that the central problems facing these countries were inadequate global supply capacity of new vaccines and insufficient domestic financing (to create a viable market for, and fund the purchases of newer more expensive vaccines) which manifested in unaffordable prices. In this regard, the Alliance was primarily set up to address between-country inequities, between the poorest and wealthier countries, and to increase the number of people worldwide benefiting from vaccination [42–44].^8^

At the time of GAVI's formation there were disparities between countries in rates of national immunization coverage with apparent divisions between strong, well-managed and well-financed programs, and weaker programs with more uncertain financing (with the latter group characterized by stagnating or declining coverage, and/or an inability to sustain gains achieved in the 1980’s through earlier concerted global immunization efforts) [45–47]. However, the discourse from that time does not suggest a strong and unanimously articulated motivation to address within-country inequities in access to immunization services. Despite relatively less emphasis on within-country disparities in documents pertaining to GAVI’s formation discussions, their importance was implicitly considered. At GAVI’s proto-board meeting in July 1999, a mission statement was adopted that underscored the importance of both achieving final outcomes to reduce VPD burden and facilitate each child’s rights to a life free from these diseases (*“To fulfill the right of every child to be protected against vaccine preventable diseases of public health concern”*). A rights-based approach implies universality and a desire to remediate inequities in the burden of VPDs faced by all individuals irrespective of socioeconomic, geographic circumstance or gender. To that extent it could be argued that within-country equity concerns were enshrined in GAVI’s stated aims from the outset – at least at the mission statement level. At the same proto-board meeting five milestones were established to monitor the effectiveness of the new immunization partnership, one of which addressed equity and specifically reductions in within-country geographic inequities (*“By 2005, 80 % of developing countries should have routine immunization coverage of at least 80 % in all districts (e.g. as measured by DTP3 and measles)”*) [48].

At GAVI’s second Board meeting which coincided with the official launch event of the Alliance (during the World Economic Forum in Davos, Switzerland), the GAVI Board discussed the policy directions and implementation mechanisms to realize the Alliance’s mission. The official record of those discussions contains two policy directions of relevance for this review. First, the GAVI Board at the time felt that selecting the ‘right’ technologies would ultimately reach previously unreached populations (“*GAVI promotes the use of new and safe technologies such as vaccine combinations and monodose delivery devices that will facilitate reaching the unreached*”). Second, through collaboration with other global health initiatives, the GAVI Board felt that the Alliance would ensure the hardest to reach would be vaccinated (“*GAVI will collaborate with other initiatives like Roll Back Malaria, African Program for Onchoceriasis Control and Micronutrient Initiative to develop effective campaign strategies to reach the most inaccessible populations*”) [49].

Regarding selection of the right technologies: There is evidence that during Phase I more Auto-Disable (AD) monodose syringes were distributed to ensure safe vaccinations (by eliminating the risk of HIV/AIDS and other blood-borne infections such hepatitis B, and hepatitis C from dirty needles). Levels of distribution of these technologies rose from 24 million in 2001 to 471 million units across GAVI countries by the end of 2005 [50]. Despite an independent evaluation of GAVI’s Injection Safety Support, which funded the switch towards AD syringes, there is no evidence to illustrate if the selection of monodose delivery devices had a positive impact on unreached or marginalized populations [25]. GAVI noted in a progress report that by the end of Phase I, of all countries approved for funds for hepatitis B vaccine, 31 (57 %) were using combination vaccines (e.g. a DTP-HepB combination, or a DTP-HepB-Hib combination) compared to just a handful prior to GAVI’s inception [50]. This suggests that during Phase I, GAVI had reduced inequities in access to vaccines between GAVI eligible countries. In theory, the convenience of additional antigens without increasing the number of injections needed to vaccinate each child during the first year of life should have prevented any detrimental effects on coverage and even helped improve coverage within countries. However, no evidence could be identified to illustrate a positive impact of combination vaccines on within-country inequities.

Regarding collaborations with other initiatives: There is evidence of GAVI Board-level discussions to foster broader collaborations with other global health initiatives – in particular with Africa Measles Campaign and the Global Polio Eradication Initiative – and these were supposed to have both advocacy and financing components [51–53]. However, GAVI’s Phase I evaluation states that only limited funding to the Africa Measles Campaign had been disbursed at the end of Phase I and deemed it unlikely that GAVI had significant impact at all during Phase I [17].

Given that at the formation of the Alliance it was recognized that many countries were characterized by stagnating or declining national immunization coverage rates, GAVI did explicitly set out to address inequities between countries in utilization of immunization services. The specifics of those efforts are discussed further below (in the section pertaining to 'Cash-based Support'). However, in aggregate the results of GAVI’s efforts to address this dimension of equity were broadly positive. The Phase I evaluation points to the fact that *“…coverage rates increased in GAVI countries during the course of Phase 1 – the DTP3 coverage rate increased from 64 % to 71 %, HepB3 coverage rate increased from 16 % to 46 %, and Hib3 coverage rate increased from 1 % to 7 %*” [17].

GAVI’s mission and milestones were heralded as the cornerstones of a new approach that intended to improve the infrastructure of vaccination [54] and address within-country inequities in immunization [55]. However, concerns were raised that GAVI’s programs would not benefit the most marginalized [56], and the absence of specific polices/programs to address these inequities (beyond selection of specific technologies and partnering with other global initiatives) suggests that at the time, the Alliance did not direct much attention or resources to this issue. Still, GAVI’s Phase I evaluation suggests some positive trends within GAVI countries over the first phase of operations (i.e. reductions in inequities in access to immunization). The evaluation assessed disparities in immunization coverage within 23 countries where more than one Demographic and Health Survey (DHS) had been conducted. The findings from the evaluators’ analysis illustrated that disparities in coverage based on urban/rural residence and gender were reduced during Phase I, with improvements correlated to total GAVI funding (for both vaccines and cash-based support). However, the evaluators found no reduction in disparities based on other important equity dimensions (e.g. mother’s educational level or birth order). The evaluators did find evidence that wealth-based disparities in immunization coverage decreased during GAVI Phase I, but this was based on UNICEF Multi-Indicator Cluster Survey (MICS) data from five countries only, and hence was not statistically significant. The Phase I evaluation also pointed to great variability on a country-by-country basis concluding that despite GAVI’s overall achievements, the Alliance still needed to develop explicit and effective approaches for facilitating support to countries with low immunization coverage and/or large internal disparities in coverage [17].

#### Phase II

In GAVI’s second phase of operations, an important step forward was the development of a comprehensive strategic plan from the outset. Covering the period 2007-2010^9^, GAVI’s strategic plan for Phase II provides a detailed view on how best to deploy GAVI’s growing resources [57, 58]. GAVI’s mission statement was amended before Phase II and enshrined in the new strategy. The revised statement was more incremental: “*Saving children’s lives and protecting people’s health by increasing access to immunisation in poor countries*” [58]. Despite removing the aim to ensure child rights, universality, and hence equity in final VPD outcomes from the mission statement, equity wasn’t entirely absent from the Phase II strategy. Both between- and within- country equity were captured within one of the GAVI operating principles that accompanied the strategy; “*Promote equity in access to immunisation services within and among countries*” [59, 60].

Improving utilization of immunization services (as measured by immunization coverage) in absolute terms and towards levels observed in higher income countries (as well as better-performing lower-income (GAVI-eligible) countries) remained a priority for the Alliance, in addition to its main goal of facilitating the introduction of new vaccines in GAVI countries [58]. GAVI's strategic goals, objectives and indicators for Phase II suggest an intention to strengthen health systems as the means to raise immunization coverage nationally with the assumption that doing so would address geographic coverage inequities within countries. However, due to a lack of consistent and reliable sub-national (district level) coverage data, the within-country equity indicator was eventually dropped and the Alliance measured its success in this phase of operations against national-level coverage estimates alone [18]. In delivering on the Phase II strategy, GAVI’s efforts were mixed. The Phase II evaluation highlights that much of GAVI’s investments to strengthen systems, and address program performance and capacity could not be measured. Where progress could be estimated (through modeled analyses), results suggested that GAVI’s efforts to increase DTP3 coverage may have only been significant for countries with initial coverage of 65–80 % and not adequate to reach out to the last 10–20 % of the unimmunized population [18].

While somewhat contradictory, the Phase II evaluation also highlighted evidence that for some GAVI countries, geographic equity of utilization of immunization services improved since GAVI funding was introduced. That is to say that the proportion of health districts reporting high coverage (those estimated to have DTP3 coverage >80 %) increased over the period of Phase II, while the proportion of low coverage districts (those estimated to have DTP3 coverage <50 %) decreased over the same timeframe among GAVI countries [18]. Neither strategies nor evidence of impact are mentioned across other dimensions of within-country equity in access to immunization.

#### Phase III

GAVI’s strategy for Phase III, was approved by the GAVI Board in 2010. GAVI’s mission remained unchanged from Phase II and as such, equity in final VPD outcomes remained absent. However, the Phase III strategy was explicit and consistent in articulating the Alliance’s focus on within-country equity in two of the revised operating principles^10^, a strategic goal-level target and key performance indicator^11^ to measure progress (to reduce inequities between socioeconomic groups in immunization coverage within countries), as well as a strategic objective^12^ [61, 62]. Most mentions of equity in GAVI’s strategic framework are not specific to either between- or within- country equity, access to vaccines, GAVI funds or utilization of immunization services.

GAVI’s Phase III strategy was augmented with an integrated 2-year Business Plan^13^ to delineate how the Alliance would support country governments to deliver on the first biennium of the strategy (2011–2012) [62, 63]. In order to improve equity in access to immunization services within GAVI countries, the Business Plan appointed WHO to lead the majority of efforts to assist countries with DTP3 coverage below 70 % and to develop coverage improvement plans (including use of “Reach Every District” -type strategies). In the second iteration of the Business Plan, covering the second biennium of the strategy, (2013–2014) the GAVI Board significantly increased both the focus and resources channeled through multilateral partners to provide technical assistance to address inequities in access to immunization services within countries. With these additional resources, UNICEF committed to focus on ten countries (Nigeria, Yemen, Congo Republic, India, Pakistan, Mozambique, Liberia, Vietnam, CAR, and Madagascar)^14^ identified by the GAVI Secretariat as having the greatest inequities in immunization coverage. The aim of UNICEF’s efforts was to identify the drivers of inequity and assist the governments of these countries to develop plans to address these inequities [64]. The GAVI Secretariat also mentioned that it had commissioned a study in Nigeria and other countries to explore issues of “*equity and trust*” given the importance of these issues in determining the effects and coverage of vaccines. It is not clear if this is the same or additional to the work commissioned through UNICEF. [65] These various country-specific initiatives and studies are currently ongoing. It is too early to evaluate the impact of GAVI’s focus to address inequities in access to immunization services within countries, but a preliminary readout of efforts to date is expected soon. When complete and published, these efforts could provide valuable insights into drivers of community demand and barriers to equity in access to immunization services in GAVI countries facing large within-country inequities in coverage.

The overall evaluation of GAVI’s results and impacts in its third phase of operations will not be available for some time. Full country evaluations to assess the impacts and challenges of GAVI support in Bangladesh, India, Mozambique, Uganda and Zambia commenced in 2013 and are expected to be completed in 2016. These may offer the first tangible results of the third phase of operations through an equity lens [28]. One limitation for the Alliance is that these five countries are far from representative of the full range of 74 countries where GAVI grants are being implemented. It remains to be seen how GAVI will garner a more fulsome and independent evaluation of its broad-ranging efforts to address inequities in Phase III.

### Vaccine priorities and New Vaccine Support (NVS)

#### Formation/Phase I

At the time of GAVI’s formation, despite the first licensures of yellow fever (YF), Hepatitis B (HepB) and *Haemophilus Influenzae* type b (Hib) vaccines in 1935, 1981 and 1986 respectively, these vaccines were virtually absent from NIPs in most low and middle income countries. As a result, there were large between-country inequities in rates of vaccine-preventable disease when comparing high income and lower income countries [42, 66–71]. Accordingly, GAVI focused its resources initially on reducing disparities in access to HepB and YF vaccines and subsequently, Hib vaccines. At the outset, there was an expectation that by the end of Phase I, GAVI would have catalyzed almost universal access to HepB [72]. GAVI’s Phase I evaluation completed in October 2008 suggests that the Alliance was indeed very successful having made huge strides to introduce HepB and YF vaccines. For example, of the 71 countries eligible for HepB and Hib vaccine support over the course of Phase 1 from 2000 to 2006, the eligible countries approved for GAVI NVS rose from 15 to 56 and 6 to 18 respectively [17].

At the end of Phase I, GAVI expanded its range of investments to include some measles campaigns in Africa – a region that had a disproportionately high measles-related mortality. While these investments could have addressed between-country inequities in disease burden, the fact that the investments occurred so late in Phase I meant that no evidence of impact on final outcomes was reported in the Phase I evaluation—or elsewhere.

#### Phase II

GAVI’s second phase of operations was characterized by expansion with more resources, initially through increased bilateral donor commitments and then by the creation and channeling of unprecedented levels of funding from the Innovative Financing Facility for Immunisation (IFFIm) [73]. These resources enabled GAVI to broaden its vaccine portfolio. In addition to continuing the scale-up of Hib-containing combination vaccines, pneumococcal conjugate vaccines (PCV) and rotavirus vaccines (RV) were added into the portfolio and NVS commitments started to cover a broader range of vaccines. The stated aims of these NVS programs were to reduce inequities between GAVI and non-GAVI countries in access to these newer vaccines, and to reduce in rates of the relevant vaccine preventable diseases [74]. GAVI’s Phase II evaluation compares use of HepB and Hib vaccines between two groups of countries: GAVI-eligible Lower-Middle Income Countries (LMICs) versus those LMICs that were not eligible for GAVI support (‘non-GAVI LMICs’). The evaluators’ analysis illustrates that at the outset, a larger proportion of non-GAVI LMICs were using these vaccines. Over the course of Phase I and particularly Phase II, the gap between the GAVI and non-GAVI LMIC groups decreased markedly [18].

The NVS programs in Phase II were also successful at reducing inequities in access to new vaccines between GAVI countries. By the end of 2009, the Phase II evaluation points out that 89 % of GAVI countries with endemic Yellow Fever had introduced YF vaccines, while 97 % and 83 % of all GAVI countries had introduced HepB and/or Hib –containing vaccines [18].

The IFFIm resources also facilitated a series of one-time investments in catch-up campaigns and replenishment of emergency response stockpiles of YF vaccines and Meningococcal A (MenA) conjugate vaccines. In addition, IFFIm resources funded one-time investments in measles, tetanus, and polio supplementary immunization activities to aid measles elimination, maternal and neonatal tetanus elimination (MNTE), and polio eradication efforts [73]. The stated aims of the elimination and eradication programs were broad – to reduce inequities in rates of vaccine preventable disease and mortality *between* countries, and specifcally within measles and MNT investment cases submitted to the GAVI Board, there is evidence of explicit intent to make use GAVI funding to address disparities *within* GAVI countries [75–77].

Modeled research illustrates that in providing access to new vaccines against diseases like rotavirus, GAVI vaccine support could benefit most the poorest groups within eligible countries because these groups are at greatest risk from infectious disease [78]. Of course, elimination and eradication offer the ultimate in equity improvement, protecting all people from specific diseases [79]. While there is extensive documentation available concerning progress over time with respect to the global polio, measles, and MNT eradication/elimination efforts, no published evidence could be found to illustrate the impact on equity dimensions of GAVI’s specific contributions. That notwithstanding, the influx of resourcing and expansion of GAVI vaccine priorities in Phase II should have positively addressed both within- and between- country inequities in vaccine preventible disease burden.

While there is limited evidence to illustrate the causal effects of GAVI investments on disease within a handful of countries for routine vaccines like Hib [80, 81], global burden of disease studies and estimates of child mortality illustrate that certain low and middle income countries – many of which benefit from GAVI support – saw marked improvements in rates of vaccine preventable disease and child mortality. These improvements extended into the first decade of the new millennium which encompassed GAVI’s phases I and II [82, 83].

#### Phase III

Through Phase III, GAVI has continued to accelerate introductions of vaccines. All GAVI countries have now introduced HepB and Hib vaccines in a pentavalent combination, all YF endemic GAVI countries have introduced YF vaccines into routine programs, and the majority of GAVI countries have introduced pneumococcal vaccines [84]. As such, GAVI has done much to address between-country inequities in access to these vaccines. However, GAVI has gone further. In 2008, GAVI developed a vaccine investment strategy (VIS) based on an assessment of the vaccine landscape that sought to identify an expanded portfolio of vaccines that GAVI should support. An Independent Review Committee of vaccine and development experts reviewed portfolios of vaccines against a series of seven criteria that included prioritizing vaccines that disproportionately addressed socioeconomic and gender inequities [85].^15^ The Board endorsed that this VIS should be based on a strategic theme of maximizing reductions in overall disease burden across GAVI countries [86]. In preparation for and during Phase III, two rounds of VIS analyses prioritized vaccines (in addition to those already in the GAVI portfolio) against the following vaccine-preventable diseases: Cholera, Human Papillomavirus (HPV), Japanese Encephalitis (JE), *Neisseria meningitides* group A (MenA), Rubella and Typhoid [87–92].^16^ Many of these vaccines prevent infections and diseases that are more concentrated in certain regions (e.g. JE in South East Asia, and MenA across the “meningitis belt” spanning the 25 sub-Saharan countries) or that primarily affect different gender and age groups (e.g. HPV causes cervical in women in later life). As such, it has been noted that by supporting these vaccines, the VIS positioned GAVI well to address inequities in disease burden between countries (i.e. *inter-regional disparities* in disease burden) as well as inequities within- and between- countries (i.e. *gender disparities* and *inter-generational disparities*) [93]. Others also point out that adding these vaccines to GAVI’s portfolio should further reduce inequities between countries in access to vaccines that have benefits in both GAVI countries and HICs (i.e. HPV vaccines) [94].

Beyond the VIS process, the GAVI Board also agreed to complement the efforts of the Global Polio Eradication Initiative (GPEI) by leveraging GPEI resources and GAVI’s experience to facilitate widespread introduction of inactivated polio vaccine (IPV) in almost all 73 of the GAVI-eligible and graduating countries by 2017 [95].

Despite the significant expansion of GAVI’s vaccine priorities, access to this wider array of vaccines at the country level has been slower than initially forecast . This is partly because of GAVI’s prior funding constraints (precipitated by the Global Financial Crisis of 2007–2008) and partly because of country readiness and national priority setting [91, 96]. In addition, issues on the vaccine supply side have conspired against rapid achievement of the impacts outlined above, including supply constraints (e.g. for Cholera vaccines), delays in regulatory approvals (e.g. for JE vaccines), and delays in development of vaccines meeting GAVI-defined specifications (e.g. conjugated Typhoid vaccines) [97]. The GAVI Board took provisional decisions to introduce Rubella, JE and HPV vaccines in 2008 yet the first GAVI-funded Rubella (catch-up) campaigns and nationwide introductions of HPV vaccines only commenced in 2013, while the first nationwide introductions of GAVI-funded JE (routine introductions) were scheduled in a handful of countries for 2014 [98, 99]. Empirical evidence to document impacts on between- and within- country inequities of GAVI’s expanded vaccine priorities in Phase III are not yet available.

Since GAVI’s first phase of operations, the Alliance has relied on an Independent Review Committee (IRC) that provides a systematic process to review new proposals for both NVS and cash-based support applications. Since 2010, the IRC has consistently recommended a greater equity focus for GAVI programs (Personal communication, John Grundy, 2015). IRC meeting reports that are available online cover the period from November 2013 to present. These reports delineate the need for improvements in NVS applications to address within-country equity and gender disparities [97, 100–103]. For example, in November 2013, the IRC reviewed 31 NVS applications across a variety of vaccines and found that 64 % of applications were ‘gender blind’ or failed to recognize that gender is an essential determinant of social outcomes impacting on projects and policies. The reports also illustrate the IRC’s consistent recommendations that GAVI strengthen application guidelines. This has resulted in GAVI guidelines stressing to eligible countries the importance of conducting more systematic analyses of inequities and gender disparities, and designing more progressive/pro-equity strategies for new vaccine programming [102–106].

With better guidelines to steer proposal development, the IRC reports highlight the progress during Phase III. The proportion of NVS and/or IPV applications that identify equity barriers has increased from 12 % in 2012, to 55 % in November 2013, and to 55 % in April 2014. Concurrently, the proportion of NVS proposals that described approaches to address identified equity barriers increased from 8 % to 41 % to 55 % over the same time period [97, 101]. Since the IRC now only reviews plans for new GAVI grants rather than progress and outcomes with existing grants, there is no commensurate evidence to illustrate the extent to which these plans have been implemented and/or resulted in more equitable outcomes.

### Supply and procurement strategies

#### Formation/Phase I

A fundamental tenet underpinning the GAVI model from the outset has been that the “*poorest country segment should have the lowest effective price*” to ensure that vaccines would ultimately be affordable [49]. At the third GAVI Board Meeting in the year 2000, this was made more clear by the Alliance partner responsible for vaccine procurement, UNICEF, in its ‘*commitment to vaccine supply and the vaccine industry*’. In this commitment, UNICEF acknowledged that it would procure vaccines for what it classified as the ‘Poorest Countries Market’ (specifying that this market segment would be defined by the GAVI eligibility criterion at the time); and that it would separately purchase vaccines for 'Other Country-specific Markets'. UNICEF requested that vaccine suppliers offer ‘*significantly discounted prices*’ for the Poorest Countries Market, and in return, UNICEF committed to not consider those as reference prices for Other Country-specific Markets [107]. In essence, this commitment proposed *equity pricing* (also known as tiered pricing) -- lowest pricing for countries supported by GAVI, and the possibility of separate (often higher) pricing for other countries. At the time, this was seen as a ‘win-win’ for all parties, facilitating the market conditions necessary to reduce the between-country inequities in access to newer more expensive vaccines [84]. GAVI’s Phase I evaluation suggests that the Alliance was moderately successful in this regard although prices did not decline as quickly as the GAVI Board had hoped [17].

#### Phase II

At the end of Phase I and in preparation for Phase II, the Alliance developed an overarching supply and procurement strategy with a particular focus on Hib- and HepB-containing vaccines [108]. This was based on an in-depth analysis of the evolution of the vaccine supply landscape that was commissioned by GAVI and conducted by the Boston Consulting Group [109]. The analyses brought into focus the complexities of the vaccine market and nuances of the interactions between pricing and a variety of other factors such as demand, competition, production costs, and contracting terms. With the focus on the markets for the two newest vaccines in GAVI’s purview (HepB, Hib) at the time, GAVI’s new supply strategy detailed a need for sufficient and sustainable supply, as well as affordable and sustainable prices, for eligible countries. However, the notion of tiered or equity pricing is not mentioned. The Phase II evaluation is silent on all between-country equity impacts of the Alliance’s supply and procurement strategy (except for the accelerated adoptions of HepB and Hib vaccines among eligible countries as described under ‘Vaccine Priorities’ above).

#### Phase III

While tiered pricing was viewed during GAVI’s initial phases of operation as a necessity and pre-requisite for reducing inequities in access to new vaccines/antigens between GAVI countries and wealthier countries, more recent discourse has provided a new and more critical narrative. To some, the global tiered pricing structure for vaccines brought about by the formation of GAVI (that guaranteed the lowest prices for GAVI countries procuring through UNICEF) has created unintended inequities between countries GAVI countries and ineligible Middle Income Countries (MICs). It is argued that many ineligible MICs that do not benefit from GAVI funding nor access to the lowest prices cannot access newer vaccines/antigens such as PCV, RV and HPV vaccines [110, 111]. According to this narrative, tiered pricing is at the heart of the problem. By contrast, advocates of tiered pricing and notably the GAVI Secretariat’s current CEO have suggested that if the mechanism were extended, it could offer a solution to reduce those same between-country inequities [112]. Evidence to conclusively justify either perspective is patchy or circumstancial. However, as noted by GAVI’s revised Supply and Procurement strategy for its third phase of operations, GAVI countries continue to benefit from the practice of tiered pricing and lowest prices worldwide [113]. Efforts to secure more consistently affordable prices for MICs (including countries that have graduated from GAVI support, and ineligible countries) has been a work in progress with some tension regarding whether this lies within or beyond the scope of GAVI [98].

### Country eligibility policies and program filters

#### Formation/Phase I

Perhaps the most explicit way in which equity between countries was considered during GAVI’s formation and first phase of operations was in the definition of GAVI’s eligibility policy. The Phase I eligibility policy was based on each country government’s ability to pay for vaccines for use within their NIP – those countries with the least ability to pay would be eligible. Average Gross National Product (GNP) per capita was used as a proxy for ‘ability to pay’ and a threshold of US $1,000 or less was applied to the World Bank’s per capita income data for the financial year 1999 to determine the list of eligible countries for GAVI’s first phase of operations [17, 114]. While documentation to explain the motivations for the policy design is sparse, the eligibility *threshold* was based at least in part on the resources available to GAVI at the time of its formation (Steve Landry: Personal communication; 2009) as well as a desire by GAVI’s architects to create an “*IDA-like*” list of countries (i.e. a list of countries similar to those that could access the World Bank’s International Development Association (IDA) loans and grants) [115].^17^

GAVI’s eligibility criterion and threshold in Phase I generated a list of 74 countries that would be eligible for GAVI support.^18^^,^^19^ Based on World Bank country classifications at the time, that list comprised 63 Low Income Countries (LICs) as well as 11 LMICs. The inclusion of LMICs from GAVI’s inception was an implicit acknowledgement: first that inequities in access to new vaccines were not neatly aligned with World Bank income classifications, and second, that all LICs and at least *some* LMICs required development assistance to alleviate disparities in immunization as compared to higher income countries.

Despite trying to improve access to new vaccines/antigens and utilization of immunization services among the lower income countries, the imposition of an eligibility threshold based on a single (per capita income-based) criterion naturally created inequities between eligible and ineligible countries. LMICs immediately above the US $1000 threshold in the financial year 1999 (e.g. the Philippines that had a GNI per capita estimate of US $1030) were unable to access GAVI support in Phase I and were probably not appreciably better able to pay for and access new vaccines than those countries just below the threshold (like Bolivia with a GNP per capita of US $970) [116].^20^

During Phase I, GAVI also instituted ‘program filters’ to determine which of the eligible countries could access particular kinds of GAVI support. Eligible countries attaining national coverage of the third dose of Diphtheria-Tetanus and Pertussis (DTP3) of less than 50 % were not permitted to apply for GAVI’s NVS grants^21^ with the exception of YF vaccine grants. The underlying rationale was that it would not be efficient to invest GAVI funds on new vaccines for countries unable to immunize at least half of their childhood populations with existing routine vaccines. Accordingly, it was felt that those countries with low immunization coverage should focus on improving immunization program performance before adding new vaccines such as HepB to their NIPs [117]. Countries with national DTP3 coverage rates of more than 80 % were not eligible for cash-based support^22^ to strengthen their immunization programs, presumably based on a notion of that these countries would be less able to markedly benefit from these small investments.

When first instituted, 14 of the 74 GAVI-eligible countries, including Timor-Leste (once it became eligible), were unable to access NVS while 22 eligible countries were unable to access cash-based support. [115, 118] The motivation driving the creation and imposition of these filters was efficiency. As a result, efficiency was seemingly traded off against between-country equity as the NVS filter in particular prevented those GAVI countries in greatest need from accessing new vaccines/antigens.

By contrast, countries applying for YF NVS grants were exempted from the DTP3 program filter on grounds that the eligible countries affected by the disease were “*the poorest of the poor… and have the weakest immunization systems …*” [119]. In a sense, the exception of YF vaccines from program filters promoted vertical equity between eligible countries by lowering the threshold to access GAVI resources (for YF vaccines) for those countries deemed to have greater needs.

#### Phase II

Prior to the start of GAVI’s second phase of operations, GAVI’s eligibility policy was updated with the latest (GNI per capita) reference data published by the World Bank for the financial year 2004. However, reliance on a sole eligibility criterion continued, and the US $1,000 eligibility threshold was left unchanged [120]. This policy update decreased the number of eligible countries to 72: four countries (thePeople’s Republic of China, Turkmenistan, Albania, and Bosnia and Herzegovina) were now above the threshold and were reclassified as ineligible for new GAVI support, and one country (Kiribati) fell below the threshold and became eligible for the first time from 2006 onwards [121]. A further update to GAVI’s eligibility policy was considered during Phase II, but the GAVI Board decided not to revise the eligibility policy largely to maintain predictability of financing support [122, 123].

The level of the DTP3 filter was left unchanged despite the fact that by 2006 only two of the eligible 72 countries (Chad and Somalia) had national DTP3 estimates according to WHO/UNICEF below 50 %.

From the perspective of between-country inequities in access to new vaccines/antigens, the revised country eligibility and the unchanged DTP3 filter policies for Phase II resulted in marginally fewer countries being allowed to introduce new vaccines; but, of those that were eligible, the vast majority were able to benefit from access to GAVI’s expanded suite of new vaccines and cash support. A modeled analysis conducted during Phase II covering a period prior to GAVI’s inception and through the middle of Phase II suggests that GAVI countries had accelerated decisions to adopt Hib-containing vaccines as compared to non-GAVI (ineligible) countries [124]. More recent evidence suggests that GAVI’s success in assisting eligible countries access to new vaccines/antigens inadvertently increased between-country inequities: Specifically, because of GAVI’s success in facilitating new vaccine introductions in eligible countries, by the end of Phase II, ineligible MICs lagged behind their eligible country counterparts [125, 126].

#### Phase III

In 2009, GAVI revised its eligibility policy once again – now considering the policy objectives, criteria, data sources and thresholds that generate the list of eligible countries. One of the most significant weaknesses of the previous policies had been the infrequency of the updates, particularly of the underlying reference data. The eligibility criteria (GNI per capita as a proxy for ability to pay for vaccines) and the threshold (GNI < US $1,000 per capita) had been defined data for the financial year 1999 and held constant throughout both Phase I and II, while the reference data for the financial year 2003 (published in 2004) had been used throughout Phase II. By holding the threshold and reference data constant for five years, GAVI had created a series of between-country inequities in access to GAVI resources when viewed through the lens of the current average per capita incomes.^23^ The Alliance was keen to avoid such inequities going forward and thus the Board approved an updated and more dynamic policy [126].

During the eligibility policy development process at the time, an issue central to addressing between- and within- country inequities was discussed; namely, whether GAVI’s eligibility policy should generate a list of the ‘poorest countries’ or a list of the countries that had the largest number of ‘poor people’.^24^ This discussion came about in large part because it was noted that within several large middle income (and ineligible) countries – notably Brazil, China, the Philippines, South Africa –a significant proportion of the population were living in poverty or extreme poverty. More often than not, substantial amounts of these populations remained unimmunized or under-immunized with GAVI-supported vaccines/antigens. In many instances the size of these impoverished and/or underserved groups within ineligible MICs was observed to be as large as- or larger than- the total populations living in many GAVI countries. It was also highlighted that several MICs that had never been GAVI-eligible lagged behind GAVI countries in terms of adoption of new vaccines/antigens.

GAVI’s eligibility policy architects for Phase III noted these valid concerns but also that there were political, operational and pragmatic reasons for GAVI to maintain an eligibility policy based on countries, rather than people as the unit of classification. The policy designers recognized that it would be difficult for GAVI to accept applications from country subunits (e.g. state governments or semi-autonomous regions) given that vaccine adoption decisions and public investments in health more generally are sovereign decisions (or at least centrally determined) and to allocate cash or in-kind resources (e.g. vaccines) to country subunits would distort fiscal federalism policies [128]. On the supply side, tiered pricing policies defined by vaccine manufacturers generally use nation-states as the unit of measure [129, 130]. Looking at GAVI operations, all of GAVI’s programs had been and still are, set up to receive applications for NVS or health systems strengthening (HSS) support from national governments. For example, each application for GAVI support requires Ministers of Health and Ministers of Finance to serve as co-signatories [131, 132]. From a pragmatic standpoint, in order to isolate countries with large sub-populations living in poverty, one would need to rely on demographic surveys (i.e. DHS, MICS). However, such survey data are not available for all GAVI countries, are conducted infrequently (e.g. at best every 2–3 years) and are conducted in different years for different countries. All this would mean that in a given year, data would not be comparable across countries nor consistently up to date. Given GAVI’s desire to have a new policy that was dynamic, such measurement challenges made reliance on survey data far from ideal as the underpinnings of an eligibility policy. Finally, GAVI’s stated mission at the time was “*Saving children’s lives and protecting people’s health by increasing access to immunisation inpoor countries*” [133]. Weighing all these facts, GAVI’s Programme and Policy Committee (PPC) confirmed that GAVI’s new eligibility policy should “(f)*ocus on poorest countries* (rather than the poorest people) *using consistent, reliable and valid indicators*” [134]. The eventual policy approved by the Board continued to rely on GNI per capita as the indicator to identify the countries with lowest average incomes. However, the US $1,000 threshold was inflation-adjusted to US $1,500 and thereafter updated annually along with the underlying reference data (i.e. to hold the threshold constant in real terms) thereby creating a dynamic list of eligible countries throughout Phase III [135, 136]. Over the course of Phase III, no new countries have become eligible, however a growing list of 16–20 countries have entered the graduation process restricting access to newer vaccines/antigens to a subset of the countries that were eligible at the outset of Phase III.

Despite maintaining the eligibility policy throughout Phase III, the debate concerning GAVI eligibility has continued to divide the GAVI Board throughout this phase of operations. This has been fueled by a variety of equity concerns. Questions have been raised about the fairness of focusing GAVI’s efforts on the poorest countries versus poorest people, the fairness of limiting access to funding for newer vaccines/antigens to GAVI eligible but not GAVI graduating countries, and the fairness of restricting access to the lowest vaccine prices to GAVI countries, but not extending those prices to ineligible MICs [137–144].

As part of the revised country eligibility policy for Phase III, the GAVI Board approved raising the level of DTP3 coverage filter for countries to be eligible to apply for new vaccines support from 50 % to 70 %. The Board agreed to raise the bar contingent on efforts being developed to improve coverage and strengthen systems in countries with DTP3 less than 70 %^25^ [135, 136]. The following justification for these changes were provided: “*On equity, this filter signals to weak performers that they need to increase coverage of traditional vaccines (and presumably reach harder-to-reach groups, who tend to be poorer) before expanding coverage to new vaccines*” [145]. The implicit assumption being that raising national coverage would address internal inequities within countries. As with earlier iterations of this policy, on grounds of efficiency the imposition of the filter continued to make it difficult for those countries with low immunization coverage to access some of the newer vaccines, exacerbating inequities between GAVI countries in access to these vaccines/antigens.

### Large countries and budget cap policies

#### Formation/Phase I

Recognizing that GAVI’s budget was particularly limited in its first phase of operations, and that not all countries under GAVI’s eligibility threshold were equal, in 2001 the GAVI Board capped resource allocations at US $40 million to the three eligible countries with the largest overall populations (China, India, and Indonesia) for the remainder of Phase I [146]. In reaching that decision, the GAVI Board recognized that those three countries had large economies and important domestic vaccine manufacturing capacity. As such, it was thought that GAVI should give these three large countries different consideration than other countries. This created another between-country equity dimension with respect to access to GAVI resources, this time between the largest three countries and other eligible countries [146].

In hindsight, these budget caps had a mixed impact on inequities in access to new vaccines, immunization services and GAVI resources more generally. The independent evaluation of GAVI’s Phase I delineates the expected total 5-year GAVI commitments by country across the various forms of GAVI support available at the time of the evaluation. The three large countries with budget caps (China, India, and Indonesia) were allocated commitments (i.e. GAVI NVS and cash-based support grants) almost equivalent to their allotted budgets (GAVI commitments to China, India, and Indonesia in Phase I are listed as US $39-40 million). In contrast, there were 13 significantly smaller countries (as measured in total population terms) with equivalent or larger allocations. These countries were Angola, Bangladesh, DR Congo, Ethiopia, Ghana, Kenya, Malawi, Nigeria, Pakistan, Tanzania, Uganda, Yemen, and Zambia. GAVI commitments in Phase I to each of these 13 countries were between US $39-155 million despite the fact that all had fewer unimmunized children, and in some cases, lower levels of infant mortality than any one of China, India and Indonesia [17]. This suggests that during Phase I, the budget caps negatively affected horizontal inequities between GAVI countries._._

Comparing how two of these large countries (China and India) used the capped GAVI budgets allocated to them illustrates that GAVI’s approach to large countries may have had varied effects on within-country inequities. A recently completed independent evaluation of the GAVI support of China's HepB vaccine introduction illustrates that GAVI support was very successful in catalyzing the vaccine’s introduction nationally [148] and addressing within-country inequities [21, 149]. Since the Government of China used GAVI resources exclusively to target the introduction in the poorest areas of the country with the worst health statistics (the 12 Western Provinces and the most impoverished counties of the 10 Central Provinces), GAVI resources were targeted progressively to address within-country inequities in disease burden, and accordingly “*eliminated socioeconomic inequities in vaccination coverage*” [150]. By contrast, in India, where much of the budget made available by GAVI in Phase I was also used to catalyze HepB vaccine introduction, the Government of India (GOI) chose to target 15 metropolitan cities and 33 districts evaluated to have a high immunization coverage (>80 %) at the time. While this approach encompassed slum populations in some cities that might be at higher risk of HepB, the focus on high coverage districts on grounds of efficiency may have exacerbated within-country inequities in access to the new vaccine, particularly affecting the rural poor [151, 152].

Separate from the budget caps, in its 2004–2005 strategic framework, GAVI indicated an intention to strengthen routine immunization and coverage in seven eligible countries with a large number of unvaccinated children (Bangladesh, India, Indonesia, Nigeria, Democratic Republic of Congo, Pakistan, and Ethiopia). This intent recognized that progress towards the global development goals at the time (e.g. Millennium Development Goals—MDGs, Global Immunization Vision and Strategy—GIVS goals) would depend on significant improvements in those counties. To help the governments of these large countries reach unreached and underserved children with new vaccines and immunization services (and hence address within-country inequities ), GAVI resources were provided to WHO and UNICEF in order to strengthen their own capacity, the capacity of other in-country partners, as well as the ability of health systems in those large countries to effectively target services to unreached populations. The intended outputs from this funding were analyses of the barriers to access, and agreed action plans that would be endorsed by national Interagency Coordinating Committees (ICCs). In addition, GAVI committed to establish specific policies to help the countries in question. GAVI documents at the time explicitly noted that these plans and policies should draw upon lessons learned from the accelerated disease control (ADC) initiatives such as those focused on polio eradication, measles control, and MNTE that were active in these large countries [153–155].

A significant weakness is that there is limited documentation available to describe the results from these efforts. It is clear that WHO and UNICEF assisted the seven large countries to analyze the barriers and possible solutions to accelerating immunization coverage and agreed action plans with ICCs by 2004. However, the extent to which these plans were implemented, and the outcomes of these activities are less clear [156]. Later iterations of GAVI’s work plan suggest that these efforts were deemed to at least be partially successful in some countries or at least no longer needed since the scope of the efforts were pared down from seven to four of the large countries where the Alliance focused its efforts [157].

#### Phase II

Early in Phase II, the GAVI Board reviewed support to large countries recognizing that the previous definition of ‘large countries’ had been based on the total population of the country rather than the birth cohort. This was despite the fact that GAVI’s support was largely directed towards subsidizing childhood vaccines. This definitional detail meant that Indonesia had been classified as a large country by GAVI, and hence had a capped budget allocation despite having a smaller birth cohort than that of several other countries not classified under GAVI’s budget cap policy as ‘large countries’ (e.g. Nigeria and Pakistan). Therefore, other smaller countries with fewer unimmunized children had accessed larger amounts of GAVI support than had been made available to Indonesia or any of the countries classified as large countries. On the basis of these between-country equity arguments, the GAVI Board removed the budget cap for Indonesia and increased the cap for the other remaining large country, India to US $100 million for Phase II [59, 158–160]. A year later, India’s budget cap was increased further to US $350 million following several rounds of negotiation with the GOI which provided the GAVI Board sufficient assurances that the GOI was strongly committed to introduce other new vaccines/antigens (i.e. Hib-containing vaccines) and to scale up domestic allocations for India’s NIP more generally [161, 162].

The relaxing of GAVI’s budget cap policies increased the ability of large countries to access GAVI resources and ameliorated between-country inequities in access to GAVI resources. However, the manner in which India, the largest GAVI country (annual births), continued to approach GAVI support (focusing GAVI resources on the best performing areas within the subcontinent) potentially continued to exacerbate within-country geographic inequities in access to new vaccines. As with the GOI’s initial applications to GAVI for HepB vaccine support in Phase I, the GOI’s Phase II applications for extended HepB support and for the planned introduction of Hib vaccine/antigen targeted GAVI resources to those districts with high routine immunization coverage. Thus, the GOI provided the new vaccines to those populations already best-served by the NIP on efficiency grounds^26^ [163, 164]. While the aim was to scale these pilot programs up across the country thus reducing geographic inequities, nationwide use of HepB and Hib in India's NIP had yet to materialize at the time of this systemmatic review.

#### Phase III

In GAVI’s eligibility policy for Phase III, the GAVI Board endorsed that a new budget cap be considered for India covering the period 2012–2015 and then revisited thereafter subject to funding availability [135, 136]. However, a new budget cap was never defined and never discussed by the GAVI Board. While no documentation could be found to support the motivations for the lapse in policy, it may be related to the fact that soon after the aforementioned Board decision on eligibility, donors committed US $4.3 billion to the GAVI Alliance – exceeding the announced target of US $3.7 billion that needed to be raised - at GAVI’s first Pledging Conference [165]. Therefore, whether by design or default, GAVI has not formally debated nor actively instituted budget caps for any eligible and large countries in its third phase of operations.

### Cash-based support for program/system strengthening

#### Formation/Phase I

Given that GAVI’s DTP3 program filter made it more difficult for countries with low immunization coverage (and often with sizable within-country inequities) to benefit from GAVI-funded vaccines, GAVI created a funding ‘window’ to which eligible countries could apply to access so-called 'cash-based support' to help finance investments in their routine immunization programs. It was hoped that this cash-based support - which eventually became known as GAVI’s Immunization Services Support (ISS) -would catalyze immunization coverage improvements by making continued funding conditional upon improvements in performance and quality immunization coverage data. In order to operate such that GAVI-funded programs were “*country driven*”, GAVI did not specify how its cash-based support should be used beyond that it was spent by/on a country’s NIP [166]. Reviews of GAVI programming during Phase I noted that the reward system employed by ISS might encourage countries to use funds to raise overall coverage, but not necessarily to address inequities in access to immunization services within countries [167–169]. One review noted that of the countries that had used ISS funds in the first year of the program, only one – Tanzania – had taken the opportunity to allocate funds to address in-country inequities, by targeting it towards low coverage districts [167]. Evidence produced later suggests that ISS had a positive effect on coverage in those countries with low levels of national immunization coverage (i.e. DTP3less than 65 % at baseline) [168], but subsequent analyses suggest that the performance-based payment system that underpinned the ISS program may have inadvertently encouraged service providers to over-report immunization coverage. This called into question the underlying data used to assess the effects on coverage and equity of this program [170]. Data challenges notwithstanding, two independent evaluations of GAVI’s ISS funding confirm mixed results [19, 20]. The second and more comprehensive evaluation conducted in 2007 and reviewing the experience of Phase I found that while the GAVI's cash-based support *did* positively affect national immunization coverage, the funding was less effective at improving national DTP3 coverage in the group of countries defined by the World Bank at the time as ‘Low Income Countries Under Stress’ (LICUS countries) as compared with the effects in non-LICUS countries. Furthermore, the evaluation found no association between ISS funding and observed improvements in geographic equity of immunization coverage within countries. As a result, one of the evaluation’s key recommendations was for GAVI to revise the ISS mechanism, emphasizing the importance of considering, among other things, equity objectives [20].

#### Phase II

One significant change to GAVI’s operations in Phase II came with the recognition that achieving improvements in immunization coverage would be dependent on having strong underlying health systems. Accordingly, and based on the findings of a series of studies conducted between 2003 and 2005 [171–173], the GAVI Board agreed to invest an initial US $500 million over a 5-year period in a new and broader form of cash-based support that became known as the HSS funding window. GAVI's HSS funds were intended for eligible countries to address system constraints ultimately to improved immunization coverage and health care delivery. Again, GAVI did not specify how countries had to spend this money, and allowed country governments to direct these resources beyond immunization programs alone [160, 174–176]. The GAVI Board set resource allocation formulae to determine the envelope of HSS funds for each country as follows: Countries with an annual GNI per capita greater than US $365 had access to an envelope of resources equivalent to US $2.50 per surviving infant per year; countries with an annual GNI per capita less than US $365 had access to US $5.00 per surviving infant per year. As noted in a paper to the GAVI Board, “*… the allocation of funds is based on number of births with special consideration for the poorest countries based on level of GNI per capita, thereby enhancing equity*” [175]. The original HSS allocation formula was designed first and foremost to ensure vertical equity in access and distribution of GAVI resources for HSS. What’s more, the original HSS guidelines produced by the GAVI Secretariat did not offer any guidance to countries on focusing HSS funding applications on pro-equity strategies to address within-country inequities [177]. Unsurprisingly, the subsequent independent evaluation of GAVI’s HSS funding through 2008 states that HSS proposals did not have a clear or visible focus to address the specific needs of underserved groups, although many of the proposals targeted sub-national bottlenecks and/or underperforming districts that included high presence of hard to reach population groups [22]^27^ [178]. The 2007 and 2009 revisions of the GAVI HSS proposal guidelines were more explicit with respect to addressing inequities in access to immunization services. They encouraged governments applying for HSS funds to consider targeting hard to reach groups and marginalized populations as well as addressing issues of within-country inequity in the provision of immunization services (including those that created gender inequalities) [22, 179, 180]. As a result, there is some limited evidence that GAVI-funded HSS grants in the latter stages of Phase II targeted sub-national barriers to access, although evidence to illustrate concrete outcomes (intermediate or final) related to these efforts are not available [180].

In 2006, the GAVI Board also approved a new pilot funding platform in order to strengthen the coordination and representation of Civil Society Organizations (CSOs) within the implementation of GAVI HSS grants and national immunization programming more generally. The platform funding was piloted in 10 countries^28^ [181–183]. It was envisaged at the outset that CSOs were, in most eligible countries, important for increasing access to immunization in general. An independent evaluation of the pilot program found that in some countries where the public sector was functioning well and was managing the principal mode(s) of immunization delivery (notably Ethiopia and Pakistan), CSOs had an important role in delivering vaccines to hard-to-reach areas or neglected population groups, thus helping to remediate within-country inequities in coverage [24]. While the approach was piloted in Phase II, an assessment of the broader (equity) impacts particularly on outcomes is not available.

As aforementioned, GAVI’s Phase II evaluation highlights that GAVI countries improved more in terms of geographic equity of access to immunization services since GAVI funding had been introduced. However, the evaluation also notes that there was no obvious relationship between GAVI’s cash-based support in Phase II and improvements in geographic equity indicators [18]. Thus, while observed improvements may have been related to the efforts of the Alliance, they cannot be attributed to GAVI funding.

#### Phase III

GAVI’s strategic plan for 2011–15 refocused HSS on immunization outcomes to “*contribute to strengthening the capacity of integrated health systems to deliver immunization by resolving health systems constraints, increasing the level of equity in access to services and strengthening civil society engagement in the health sector*” [61, 144]. Thus, at the strategic level, equity and the importance of CSOs was made far more explicit as was the fact that HSS investments ultimately ought to demonstrate a results chain back to immunization outcomes.

Building on the findings from the HSS evaluation conducted during Phase II [22], GAVI published guidance to help countries applying for HSS funding to consider gender-related barriers to access (i.e. social and cultural norms that influence men’s and women’s roles and that ultimately impact access to immunisation services) [184]. GAVI noted that HSS funds could be used to identify– through special studies or investigations – gender-related barriers in the national health system and within the immunisation services. HSS funds could also be used to remove such barriers through capacity-building of health services and community staff and special interventions. No evidence could be found to demonstrate the extent to which GAVI's HSS funds have subsequently been used to address these inequalities and disparities or the extent to which HSS investments have resulted in reducing such barriers.

In November 2011, the GAVI Board decided that GAVI’s HSS funding would be consolidated into a single window (i.e. no separate windows for HSS and ISS, nor separate applications for funds directed to governments versus to CSOs). The revised HSS window required that all countries be approved for the HSS performance-based funding (PBF) component. As with GAVI’s HSS funding in Phase II, the total funding envelope for each country (referred to as ‘country ceiling’) continues to be based on the country’s GNI per capita and total population—i.e. the amount of cash-based support is still determined with the aim of ensuring equity (between eligible countries) in access to GAVI resources [137, 185]. However, in addition to a lump sum, PBF payments are determined based on progress against the national DTP3 coverage level at baseline. For countries with national DTP3 coverage <70 % or 70–89 % at baseline, performance payments are dependent on coverage improvements in DTP3 and the first dose of measles containing vaccines (MCV1). For countries with national DTP3 coverage ≥90 % at baseline, performance payments are split between maintaining national DTP3 coverage levels and minimizing within-country geographic inequities in coverage (i.e. specifically ensuring that 90 % of districts have ≥80 % DTP3 coverage) [186, 187]. This is the first and only explicit example of a GAVI resource allocation mechanism that is designed to channel resources to eligible countries based on grounds of within-country inequities in utilization of immunization services. Despite this decision taken in 2012, GAVI has yet to publish any reports summarizing the scheme’s resultant impacts on coverage and equity. However, one case study has been published delineating the effects of GAVI’s PBF-based HSS in rural Cambodia. This notes that the GAVI support did bring about an increased volume of services to underserved rural areas along with strengthened financial and operational management. However, the authors note that the PBF scheme did not help ensure high quality of these additional services [188].

GAVI’s IRC reports highlight the limitations of HSS applications submitted during Phase III with respect to inequities and gender disparities within countries. Notable deficiencies flagged by the IRC include: limited identification of underserved groups and the equity/gender barriers that hinder the groups’ access; limited articulation of strategies to address these barriers and/or monitoring frameworks to track progress to ameliorate the situation; lack of CSO involvement in development of HSS proposals to ensure underserved are not overlooked; insufficient consideration of how CSOs can help address disparities particularly in conflict situations; insufficient consideration of how integration with other programs can help address inequities [97, 100, 102, 103]. Given that the IRC often requests resubmissions or clarifications before recommending the GAVI Board to fund country proposals, the independent reviews do likely sharpen country plans to address inequities and gender disparities within countries using HSS funds. However, as aforementioned with IRC reviews of NVS proposals, there is no commensurate evidence available as yet to illustrate the equity outcomes from GAVI’s investments.

While not directly about GAVI’s PBF scheme nor specifically about GAVI’s HSS investments in Phase III alone, one ethnographic case study of the evolution of GAVI’s role in HSS is considerably more critical. The author states that the most important effect of the creation of GAVI’s HSS window has been to strengthen the power of vertical global health initiatives like GAVI in defining the global health agenda. Furthermore, the paper suggests that GAVI’s expanded role in HSS has legitimized some of the practices (e.g. private financing, a narrow focus on measurable health outcomes) that the author argues have contributed to decimating poor countries’ health systems [189]. While the author does not explore how GAVI’s efforts have helped or hindered between- or within- country inequities in access, the inference is that GAVI’s involvement in HSS has not been wholly positive.

### Vaccine introduction grants and policies

#### Formation/Phase I

Early experience with vaccine introductions during Phase I led GAVI to provide countries with financial resources to facilitate introductions (e.g. in order to upgrade immunization delivery infrastructure, such as replacing an ageing cold chain or training new health staff). Specifically, eligible countries were provided with another form of cash-based support, as a one-time cash grant of US $100,000 at the time of introducing each new GAVI-supported vaccine [190]. In this regard, resources to support vaccine introductions was shared equally in absolute terms among all countries planning vaccine introductions. A review of experience with vaccine introduction grants in Phase I recognized that the lump sum award was *“…not an equitable way to support new vaccine introduction activities. Given widely varying population sizes in GAVI-eligible countries, not all countries benefited equally from this cash award… Allocations ranged from a maximum of US $19.0 per infant in Sao-Tome and Principe, to a minimum of less than US $0.01 per infant in Pakistan*” [191, 192].

#### Phase II

To address the inequities (between small and large GAVI countries) in access to GAVI vaccine introduction grants (VIGs) that were inherent within the original policy, the policy was revised policy. The new policy stipulated that countries should be provided an amount (US $0.30) on a per infant basis with a minimum absolute award value (US $100,000) for the smallest countries . Recognizing that the full extent of introduction costs was unknown – particularly for newer vaccines against rotavirus and pneumococcal diseases that GAVI was planning to support – the figure of US $0.30 per infant was derived based on an adjusted US $100,000 award divided by the median number of infants in the GAVI countries (excluding India).The minimum award was set at the levels of the grants in Phase I (i.e. US $100,000) [192].

It would not be until GAVI’s next phase of operations when additional data on estimated costs of introduction would become available and the sufficiency of the VIGs could be tested. With hindsight, GAVI’s VIGs were likely insufficient to cover needs (G. Gandhi, P. Lydon, E. Furrer, H. Saxenian, A. Nguyen: *Costs of Introducing Childhood Vaccines in Low and Lower Middle Income Countries: Inputs for GAVI Policy on Introduction Grant Support to Countries*, in press). Funding constraints may have hindered many countries’ ability to harness the opportunity of a new vaccine introduction to improve vaccination coverage and/or reduce within-country inequities in access to vaccines, although existing evidence is unsufficient to confirm this hypothesis [193–195]. In any result, GAVI’s guidelines for new vaccine introduction applications in Phase II were largely silent on the need for pro-equity programming in new vaccine introductions [104].

#### Phase III

GAVI updated its VIG policy in 2012. As with the VIG policy update in Phase II, the policy design continued to ensure the equity of access to GAVI VIG resources among eligible countries based on the size of the country. However, with more information available on the costs of new vaccine introductions ([196], (G. Gandhi, P. Lydon, E. Furrer, H. Saxenian, A. Nguyen: *Costs of Introducing Childhood Vaccines in Low and Lower Middle Income Countries: Inputs for GAVI Policy on Introduction Grant Support to Countries*, in press)) as well as the operational costs associated with GAVI-funded campaigns [197], GAVI increased funding for routine vaccine introductions from US $0.30 to US $0.80 per child in the birth cohort with the minimum still set at US $100,000. GAVI also increased the funding for operational costs of campaigns that it supported from US $0.30 to US $0.65 per targeted individual [198]. While not the driving motivation for the change in levels of GAVI investments, the increase in GAVI resources for VIGs and campaign operational costs may have made it easier in Phase III for national programs to ensure new vaccines and campaigns are delivered to a greater proportion of birth cohorts/target populations, including the harder to reach. Once again there is as yet no evidence to link the change in the magnitude of GAVI's investments to changes in coverage (equitable or otherwise) within eligible countries.

### Financial sustainability (Co-financing) policies

#### Formation/Phase I

Early in Phase I, the GAVI Board articulated a desire for eligible country governments to achieve “*financial sustainability*” 29 and to create fiscal space “…*such that governments can assume the costs of the vaccines and pursue other immunization delivery goals including improved equity*” (Although the notion of equity here was not further elaborated, one can assume that the GAVI Board intended for governments to use greater fiscal space to pursue equity improvements within their own countries) [146, 199]. At the time, GAVI provided vaccines free of charge to eligible countries with the expectation that by the end of Phase I, financial sustainability would be achieved by all countries facilitating the end of GAVI support. This was based on the assumption that the additional demand generated through GAVI financing would lead to sufficiently low vaccine prices that would be affordable to developing countries once GAVI support ended. However, as became apparent by the end of GAVI’s first phase of operations, the GAVI Board’s expectations on price declines and the sufficiency of domestic financing proved to be overly optimistic, particularly given the shift towards combination vaccines where vaccine markets were characterized by less competition at the time [17]. Given this, it seems logical to conclude that the additional fiscal space expected to enable equity improvements may not have materialized either although no evidence from the literature review could be found to support or refute that supposition. For the purpose of this review, it is assumed that during Phase I, GAVI had neither a clear framework (despite the above-mentioned objective) nor any evidence of impact across any dimensions of equity as related to or facilitated by financial sustainability.

#### Phase II

Since vaccine prices had not declined to the levels anticipated by the end of Phase I, the GAVI Secretariat and Alliance partners discussed how to delay the termination of GAVI financing, how to help country governments to identify/create the required fiscal space to finance vaccines, how to improve access to their immunization programs, and how to accelerate the vaccine price declines themselves [108, 200–202]. These broad-ranging discussions laid the groundwork for GAVI’s co-financing policy, adopted in 2006. The policy required eligible country governments to take responsibility for a portion of the costs of routine new vaccines that had been financed by GAVI. GAVI’s co-financing policy is mentioned here not because there is evidence that it directly impacted inequities in access to immunization services, but because the architects of the policy mechanism incorporated (vertical) equity considerations when defining co-financing levels: Eligible countries were grouped according to their ability to pay using a variety of proxy criteria.^30^ These country groupings defined the co-financing requirements for new vaccines adopted with support offered during Phase II [203]. The co-financing policy groupings implicitly affected a more equitable allocation of GAVI’s resources in Phase II for new vaccine support. Countries with lower national incomes, and/or greater fragility received a greater share of GAVI resources *on a per dose basis*, as compared to wealthier and more stable countries. The literature and recent independent evaluations to date have been silent on the equity impacts of this policy [27].

#### Phase III

GAVI also updated its co-financing policy in 2010 with the new policy taking effect in Phase III. While the Alliance acknowledged the central importance of health financing more broadly to ensure strong health systems and immunization programs, the updated co-financing policy sought to maintain its approach to defining co-financing levels for GAVI-funded vaccines on an equitable basis. Under this iteration of the policy, co-financing levels were based on average national per capita income alone as a proxy for country’s ability to pay [63, 204, 205]. This maintained and refined GAVI’s between-country vertical equity approach in allocations of GAVI resources for new vaccine support such that on a per dose basis, lower income countries (as defined by GNI per capita) paid less, and accordingly receive a greater share of GAVI resources than wealthier countries. In practice, as noted by a recent evaluation, the policy has not been applied consistently across vaccines with some vaccines exempted (e.g. HPV demonstration projects, JE and Rubella campaigns) [27]. This may have created implicit financial incentives to prioritize some vaccine programs over others, and inadvertently albeit very marginally, affected between- and within- country inequities in disease burden. However, no evidence could be found to substantiate or refute this supposition.

### ‘Fragile States’ policies

#### Phase I

GAVI had no specific policies designed to support fragile states^31^ nor did any of the policies in GAVI’s first phase of operations have specific features to address the needs of such countries. Despite this, the Phase I evaluation interrogated the differences in the likelihood and amount of GAVI support received among different types of countries including those classified by the World Bank as being a LICUS versus non-LICUS country.^32^ Overall, the evaluation found that GAVI’s policies and requirements for country support resulted in relatively more funding per infant allocated toward three non-exclusive groups of countires: LICUS countries, those countries with lower DTP3 coverage rates at the inception of GAVI, and those countries with lower average per capita incomes. However, the evaluators also found evidence that LICUS countries were not as well able to make good use of the funding, and worse still found that GAVI funding had essentially no measurable impact in countries with an ongoing conflict (i.e. within a subset of LICUS/fragile states). The evaluators summarized that the design of GAVI support in Phase I “*may not be appropriate for LICUS countries, which tend to have lower rates of uptake and achieve lower results*” [17].

#### Phase II

Building on the findings from the Phase I evaluation, the GAVI Phase II Strategy proposed to measure GAVI’s progress to improve coverage in fragile states and Countries with Large numbers of Unimmunized Children (CLUCs) by assessing changes in geographic inequities in immunization coverage (the proportion of the populations in these countries that had ‘increased or sustained DPT3 and measles coverage at national and district level’) [153]. There was little in the way of GAVI-specific policies and programs specifically designed to address geographic inequities in access to immunization services within countries and especially for the subsets of countries described as Fragile States or CLUCs beyond (i) encouragement to use GAVI cash-based support to address specific areas or groups of the population; (ii) funding to WHO and UNICEF for technical assistance (TA) to assist countries to increase and sustain their coverage at high level; (iii) funding to WHO and UNICEF specifically to continue strengthening their own and national capacity in CLUCs [155]. On all these fronts, evidence of the outcomes associated with these efforts is sparse and somewhat inconsistent. What information is available suggests that the Alliance as a whole did not make much progress. With respect to the TA provided by WHO and UNICEF, a significant part of Alliance partner efforts seems to have focused on the definition of corrective actions based on ‘Reach Every District (RED) strategies’ and their implementation [206–208]. While GAVI’s Phase II Evaluation doesn’t comment specifically on the effectiveness of those efforts, separate evaluations of RED strategies in India and the African region suggest improvements in the quality of services and/or levels of immunization coverage [209–211]. These evaluations however say little about the impacts of RED strategies to address inequities in access to immunization. The evaluations struggled to attribute effects observed to the specific RED interventions and some of the evaluations draw on data that relates to GAVI’s first- rather than second- phase of operations, and draw upon work not solely funded by GAVI. Reports by the GAVI Secretariat documenting annual work plan achievements for 2007 are somewhat contradictory with respect to CLUCs. One states that “*By 2007, all seven CLUCs (Nigeria, Pakistan, India, Ethiopia, Sudan, DRC, Indonesia) had begun to implement Reach Every District (RED) planning, an approach that aims at improving the organisation of immunisation services so as to guarantee sustainable and equitable immunisation for every child. Five out of the seven CLUCs had improved their routine DTP3 and measles coverage by 10 % over 2005 levels*” [182]. Another report from around the same time explains *“…substantial changes have taken place in thinking regarding support to fragile support to fragile states and Countries with a Large Number of Unimmunised … While initially the intent was to provide these countries with additional technical and funding support to overcome their features through the possible expansion in the funding windows, the current thinking sees these features are ‘modifiers’ to the existing GAVI application and reporting rules as well as modifiers to funding allocations*” [212]. The GAVI’s Phase II Evaluation points out that the Alliance failed to formalize criteria to define a list of CLUCs or fragile states. Hence the Alliance’s added value in this important set of countries could not be measured [18].

The Alliance did attempt to develop a specific strategy for fragile states in Phase II agreeing on a definition of fragility based on the World Bank definition [183, 213]. As the GAVI Board came to consensus on the use of that third party definition, a World Bank independent evaluation of the Bank’s own support to LICUS noted that the Bank would need to reexamine the appropriateness of the criterion to identify LICUS given the shortcomings of the indicators and criteria used [214]. This stymied GAVI’s efforts to develop a fragile states policy in Phase II.

Given that GAVI did not develop and implement a fragile states policy, GAVI was unable to tackle how funding applications or application reporting requirement should be altered to address the unique circumstances in fragile states and CLUCs. As a result, it seems reasonable to summarize that the Alliance did not find a way to address the inequities in access to immunization within fragile states and CLUCs, nor between these countries and other countries during its second phase of operations.

#### Phase III

Early in Phase III, the GAVI Board adopted a policy on fragility and immunization [215]. Noting the many lists and definitions had been developed to identify and/or categorize fragile states, and that 77 % of GAVI countries could be considered as 'fragile' on at least one of these lists, the Board approved a country-by-country approach for a subset of countries facing particular challenges rather than developing a policy centered on a specific fragile states definition. The objectives of this tailored approach policy are to protect immunization systems and existing GAVI support in case of emergency events, and to improve vaccination coverage in a subset of countries with particularly "*challenging circumstances" * (e.g. not applying for, accessing or utilizing GAVI support, CLUCs, countries with national DTP3 coverage levels <70 %, or countries with “*equity concerns*”) [216]. The policy defines countries with equity concerns as being those countries with significant subnational, socioeconomic or gender differences in immunization coverage defined by the following criteria: greater than 50 % of districts reporting DTP3 coverage <50 % (to measure subnational disparities); where the difference in DTP3 coverage between the lowest wealth quintile and the highest wealth quintile is greater than 20 % points (to measure socioeconomic disparities); and/or where the odds ratio confidence interval (female versus males) in sex-disaggregated coverage does not include the value 1 (to measure gender inequalities) [217]. In these sets of country-specific circumstances, the policy enables GAVI to make additional resources available to address the situations, tailor support, re-program cash support, channel cash-based support and/or vaccines through in-country Alliance partners (e.g. WHO, UNICEF) or through non-state actors (e.g. CSOs). The policy also offers flexibilities in the application of GAVI policies (e.g. co-financing) [216]. While the policy implementation is in its infancy, it offers significant potential with respect to reduction of within-country inequities in access to immunization services in a subset of GAVI countries.

Beyond the country-by-country policy, in Phase III, GAVI has acknowledged a need to sharpen its approach to address the needs of fragile states in two additional ways. Firstly, GAVI has provided modest funding to the CSO constituency through Catholic Relief Services (CRS). With this funding, CRS is supposed to facilitate the development of national networks of CSOs in order to promote their engagement in the policy dialogue, service delivery, and more specifically in the design and implementation of the HSS grants to support immunization. In theory, all of these measures could contribute to equity improvements within a country [218]. Secondly, and related to the above point, GAVI’s IRC has highlighted the interrelated issues of conflict and inequities within GAVI countries since populations affected by conflict are often displaced, not well-served by government programs, or worse still 'invisible' to the government-led programs. Accordingly, the IRC has underscored the need for GAVI to rethink its modest support and light-touch approach to CSOs (given the importance of CSOs in addressing the needs of populations affected by conflict) [100, 102, 103]. While GAVI has yet to respond to the IRC’s recommendations in this regard, the IRC does now regularly profile the extent to which CSOs have been involved in NVS and/or HSS proposal development and planned program efforts [97, 100–103]. No evidence is currently available to demonstrate whether these efforts have resulted in positive impacts on inequities within GAVI countries and especially those characterized by fragile contexts.

### Gender policy

#### Phase I

In GAVI’s first phase of operations, it had no specific Gender Policy nor any programs focused on addressing gender disparities. Nonetheless, as aforementioned, the Phase I evaluation noted that disparities in immunization coverage based on gender were reduced during Phase I and changes were correlated to GAVI funding [17].

#### Phase II

On the basis of an independent report commissioned by the GAVI Secretariat [219], in 2008, mid-way through Phase II, the GAVI Board approved GAVI’s first Gender Policy to address gender inequalities in access to and coverage of immunization within GAVI countries. The policy encouraged countries and partner organizations to support the generation, reporting and analyses of new gender-related evidence with respect to immunization (e.g. gender-disaggregated coverage data). The policy also urged countries to build on such data and to strive for a gender-sensitive national immunization policy , and to more broadly encourage gender equality in health [86, 220]. However, the original independent report that provided the foundations of GAVI’s Gender Policy was poorly received and the policy was not widely supported across the Alliance. This lead to further assessments and guidance by the WHO’s Initiative for Vaccine Research (IVR) and its Strategic Advisory Group of Experts (SAGE) on gender equality in immunization [221, 222]. These assessments suggested that childhood immunization programs aren’t broadly characterized by gender disparities in coverage. Given the initial disagreement regarding the evidence that informed GAVI’s Gender Policy, it is no surprise that GAVI’s Phase II Evaluation is silent about GAVI’s impact on gender inequalities during its second phase of operations [18]. A more recent and specific evaluation of GAVI’s Gender Policy that assesses the policy’s impact within Phase II and beyond suggests that the main achievements of the policy have primarily benefitted GAVI Secretariat staff and influenced GAVI’s own governance structure (due to more emphasis on gender equality within the GAVI Secretariat and on the GAVI Board). The evaluation noted that significant effects within GAVI countries - in terms of reductions in gender disparities in immunization coverage where they exist, and more importantly efforts to address gender-related barriers to access - had yet to be fully realized [23].

#### Phase III

Building on findings from the HSS evaluation [22] as well as the Gender Policy evaluation [23], GAVI revised its Gender Policy in 2013. The revised policy shifted and broadened the programmatic focus of GAVI efforts related to gender beyond gender-disaggregated immunization coverage data. The new policy underscored: the importance of analyzing gender-related barriers to access and utilize vaccination, and strategies to overcome said barriers in NVS and HSS proposals. The policy additionally emphasized the need for GAVI to integrate gender aspects in its program guidelines, application materials, and application review criteria. Finally, the new policy committed GAVI to increase accountability for gender-related results throughout the Alliance, including in the review of program performance [223]. The impact of this new policy on outcomes has yet to be documented. Through the recent IRC reports, it seems that there has not been much progress in the extent to which country applications have identified, or sought to address, gender-related barriers. The proportion of applications that identify gender-related barriers was 12 % in 2012, 24 % in November 2013, 13 % in February 2014, and 9 % in April 2014. The proportion of applications that propose strategies to address gender-related barriers identified are lower still [97, 100, 101].

### Program prioritization

#### Phase III

Following the Global Financial Crisis of 2007–2008, and with growing demand from eligible countries for GAVI funding, the Alliance came to realize that unless donors significantly increased their contributions, and program efficiencies could be found, GAVI would be unable to fund new programs from 2012 onwards [224, 225]. A pilot mechanism to prioritize among country funding applications was developed for use only in the instance that resources became insufficient to fund new programs [226]. One of the six objectives of this mechanism was to “*Distribute GAVI’s resources more equitably among countries*”^33^. In order to achieve this between-country equity objective, the mechanism proposed the use of a simple rule: only one new vaccine proposal per country should be considered for funding in each proposal round [226]. It was also noted that other criteria to rank vaccine applications – such as health impact and per capita income – would also address between-country equity dimensions by prioritizing country applications with the greatest ability to avert deaths per 1000 vaccinated and with the least ability to pay for the vaccines [227]. The mechanism did not explicitly consider prioritization (or de-prioritization) of proposals based on levels of within-country inequities since GAVI neither wanted to reward countries with large internal inequities in immunization coverage, nor penalize countries with more fairly distributed and accessed immunization services. To date, the prioritization mechanism has not been employed to inform funding decisions given the generosity of GAVI's donors [165]. As a result, the mechanism’s effect on within- and between- country equity has been neutral.

## Discussion

### Equity of opportunity: Access to new and underutilized vaccines

GAVI’s formation and subsequent phases of operations have been characterized by a drive to address the inequities in access to vaccines/antigens between higher-income (ineligible) countries and lower-income (GAVI-eligible) countries. Evidence suggests that the Alliance has been most successful and most consistent across its phases of operations to date in addressing these particular inequities. Those successes have been primarily mediated through GAVI’s Vaccine Priorities, Country Eligibility, long-term and secure funding that GAVI has committed (NVS grants), and the Alliance’s Supply and Procurement strategies to harness the lowest vaccine prices.

GAVI’s focus on inequities in access to vaccines can be traced back to the situation observed at the time of GAVI’s formation; namely, the long lag between new and underutilized vaccines being made available to higher-income countries as opposed to lower-income countries. Consideration of the actors involved in the formation of the Alliance provides some deeper insights. GAVI’s first few years were funded mainly by philanthropic institutions (most notably, the Bill & Melinda Gates Foundation and the Rockefeller Foundation) and a small number of sovereign donors. ‘Global-level’ stakeholders from these funders and founding partner multilateral agencies (UNICEF, WHO and the World Bank) drove strategy and policy discussions [228]. Thus, a focus on addressing inequities between higher-income countries and lower-income countries may reflect the viewpoint of these stakeholders at the time—a view of the ‘developing world’ from the ‘developed world’. That said, many of the policy/funding levers to address the between-country inequities in access to vaccines – most notably increasing funding availability, and pooling that funding and procurement to facilitate access to more affordable vaccine prices – are levers that global stakeholders in particular have been best positioned to operate.

In trying to address inequities in access to vaccines between lower-income countries and higher-income countries, over time GAVI has reduced inequities between lower income countries themselves. While this was not an explicit goal of the Alliance during any of its phases, this has been an important and lasting result.

### Equity of opportunity: Access to and/or allocation of GAVI resources

Throughout each phase of its operations, like most global funding mechanisms, GAVI has had to optimize resource allocation decisions within its semi-fixed budget constraint. In terms of resource allocation mechanics and program policies, GAVI focused almost exclusively on between-country equity concerns. With the exception of the Country Eligibility policy, this focus dealt with equity between GAVI countries alone. In most instances, policies dealing with access to GAVI resources have been driven by vertical equity concerns: they sought to apportion greater resourcing to greater needs. However, the measures of need have often differed from policy to policy. For Country Eligibility and Co-financing, needs have been primarily been defined in terms of ability to pay for vaccines; for Cash-Based Support (depending on the specific program) needs have generally been determined by country size, average wealth, and NIP performance; for VIGs, needs have been based on country size. It is also worth highlighting two new/revised policies unveiled in Phase III—the Fragile States policy (Country-by-Country Approach), and the PBF-focused HSS/Cash-Based Support: These are the first GAVI policies that explicitly allocate greater GAVI resources towards greater need but where need also includes within-country inequity considerations.

One deviation from a vertical equity approach has been the implementation of Country Budget Caps in Phase I and II for a handful of large countries. While country size was the motivating factor for use of budget caps, the magnitude of the country-specific caps themselves appear arbitrary at least as compared to relative need. Evidence on how GAVI’s two largest (formerly) eligible countries – India and China – targeted their limited allocations illustrates the inherent conflict between equity and efficiency in health resource allocation decisions [229]. GAVI itself has made similar trade-offs between equity and efficiency through its employment of the DTP3 filters in the eligibility policies. Recent modeling evidence pertaining to a cadre of child health interventions including vaccines, challenges the notion that addressing inequities is not cost-effective. The research illustrates that when relative disease burdens are adequately taken into account, targeting the most deprived areas may actually be as, if not more, cost-effective than targeting well-performing areas [230].

Perhaps the most controversial of GAVI’s distributive policies has been the eligibility policy. While a necessity for any funding mechanism, by applying a principle of vertical equity and continuing to enshrine a country as the unit of eligibility (as opposed to a country subunit such as a region or health district), GAVI’s eligibility policies have naturally created ‘haves’ and ‘have nots’, and has impacted between-country equity in both positive and seemingly negative ways. Evidence suggests that for some vaccines/antigens at certain time-points, many of the countries that minimally exceed GAVI’s eligibility thresholds have had slower rates of adoption of new vaccines than many GAVI countries. This review alone cannot answer the extent to which this inequity is perpetuated by a lack of access to GAVI resources and favorable pricing versus other factors at the government level and beyond GAVI’s control (e.g. political will to introduce a new vaccine, accompanying government investment in immunization services). Further research to assess the nature of inequities between GAVI and non-GAVI countries has been undertaken -- led by WHO -- in the development of a 'Shared MICs Strategy' to address the immunization needs of MICs.

Finally, since GAVI’s major donors have been a strong voice in the governance of the Alliance, the scope and design of GAVI’s strategies, policies, and programs may be strongly influenced by resourcing considerations. This may have been particularly important during GAVI’s third phase of operations when much of the strategy, work plan, program and policy re-design coincided with the international financial crisis. This was a time when many overseas development assistance (ODA) budgets among GAVI’s sovereign donors came under more intense scrutiny from domestic taxpayers and politicians [231, 232]. That broader backdrop may have colored the strategic and policy choices taken by the Board at that time and may have affected the extent to which GAVI invested in certain pro-equity strategies.

### Equity of (intermediate) outcome: Utilization of immunization services

While GAVI’s overriding goal has been addressing inequities in access to vaccines, at formation there was a secondary priority to address inequities between countries in utilization of immunization services. This was driven by the fact that many lower income countries at the time were characterized by stagnating or falling national immunization coverage. GAVI’s effects on reducing these inequities and raising national coverage rates in GAVI countries is well understood. Since most ineligible countries have had significantly higher coverage rates than the majority of eligible countries, observed improvements among GAVI countries would naturally reduce these between-country inequities. The evidence across GAVI’s phases of operations illustrates the most rapid improvements in coverage in Phase I although these improvements appeared not to benefit countries characterized by some form of fragility as much as other countries. In later phases, evidence on reductions in immunization coverage/utilization inequities has been patchier – and immunization coverage data illustrate that there are still many children who are not fully immunized. In aggregate, the trends suggest that GAVI’s efforts to address inequities between countries in utilization of immunization services have addressed the ‘quick wins’ while the hardest to reach populations often in the countries with weakest systems and poor governance have at least until recently, remained underserved.

Addressing within-country inequities in utilization of immunization services - particularly in GAVI’s first two phases of operations - was not a central goal for GAVI. This relative lack of prioritization is likely related to three factors: (i) vaccines themselves are considered to be among the most equitable interventions; (ii) at GAVI’s inception coverage rates in most GAVI countries were so low that targeting underserved/marginalized/unreached communities was not a priority; (iii) data systems wouldn’t have allowed for such targeting in any case. This author will discuss each of this points in turn.

Vaccines are generally regarded as highly equitable interventions (e.g. targeting boys and girls alike) and, most importantly, capable of conferring population-wide herd immunity benefits against VPDs at high enough levels of coverage. For much of GAVI’s first two phases of operations, within-country equity considerations were felt to be subsumed within efforts to raise national coverage levels – after all, 100 % national immunization coverage implies zero inequities within countries. GAVI’s leadership at the time implicitly assumed that provision of cash-based support (ISS, HSS) to raise national immunization coverage levels would lead to reductions in subnational disparities. The majority of GAVI’s targets and indicators that explicitly consider immunization coverage have almost always been based on national average estimates (rather than subnational measures that consider pockets of inequity in access to- and utilization of- immunization services *within* countries). This reliance on national averages resonates with the approach pervasive throughout the global development agenda over the past 10 to 15 years and one that has characterized the MDG efforts. It is now recognized that reliance on national averages alone to measure global development progress has been a significant weaknesses of the ‘MDG approach’. Recent evidence illustrates that across many countries, reliance and focus on national averages alone has led to an exacerbation of inequities in access [10–14]. At some point, one has to acknowledge and focus efforts to reach underserved/marginalized/unreached communities explicitly -- especially when those communities are separated from proximity to better served communities and unlikely to avail from herd immunity benefits.

At GAVI’s inception coverage rates in many GAVI countries were so low^34^ that reducing the total numbers of unimmunized children may well have been an acceptable primary focus rather than prioritizing underserved/marginalized/unreached communities. However, this assumption overlooks the fact that those communities often face the greatest risks of disease and malnutrition (and as such have the greatest chance of benefiting from immunization) as compared to other communities.

In the early years of GAVI, data systems to determine performance of immunization programs nationally - let alone sub-nationally - were very weak in eligible countries. With inadequate data, designing programs to target and ameliorate sub-national inequities in access to- and utilization of- immunization services may have been too difficult. However, the fact that systems are weak is a poor justification to not attempt to help those in greatest need.

Perhaps in response to the points above, more recently, GAVI has increasingly sought to address within-country inequities in utilization of immunization services in GAVI countries. GAVI’s third phase of operations has placed more emphasis on addressing within-country inequities within the strategy, business plan, Fragile States policy, and the inclusion of subnational performance-based aspects of GAVI’s revised HSS window. Most importantly, within Phase III, GAVI has provided clearer guidelines to countries applying for NVS and HSS grants on how to ensure inequities and gender disparities are identified and addressed. The latter owes much to the critical appraisal of country applications by GAVI’s IRC and the IRC’s recommendations on how GAVI should continually improve its guidelines and policies to better address evolving priorities in immunization programs. Aside from the IRC’s recommendations, the drivers for the above-mentioned shifts may reflect the fact that GAVI has been so successful at reducing between-country inequities in access to vaccines/antigens – especially for HepB, Hib, YF vaccines and to some extent PCV– that GAVI can now bring into focus what have historically been a secondary or tertiary priority. Looking beyond GAVI, the discourse surrounding the global health and development agendas has moved from a comparison of ‘developed’ and ‘developing’ (or wealthy and ‘resource-poor’) countries to a more people-centric approach that demands all development efforts focus on individuals, including the marginalized and underserved, no matter where they reside [6, 8]. Indeed, GAVI’s multilateral and CSO partners, and most notably UNICEF, and Save the Children, have made reaching unimmunized populations a mantra in global immunization discussions. The Alliance may well be responding to those prevailing development trends and the high-level advocacy from constituents within its own ranks.

### Equity of (final) outcomes: Impact of GAVI priorities and investments on VPDs

As expected, the evidence for GAVI’s impact on final outcomes in general - let alone equity dimensions of these outcomes - is most scant. The majority of GAVI’s impact on reducing inequities in VPDs between countries has been mediated by acceleration of rates of introduction of new vaccines combined with the steady expansion of the portfolio of GAVI-subsidized vaccines. The expansion of VPD priorities means that GAVI is now able to address an increasing proportion of the burden that predominantly affect lower-income countries. Most of the available evidence characterizing the impacts on these equity outcomes are theoretical or modeled. However, the evidence suggests that GAVI investments should in the longer term enable reductions in inter-regional, inter-generational and gender-related inequities in VPD burden.

There are specific examples of the magnitude and speed of GAVI’s impact on final outcomes through facilitation of vaccine introductions within GAVI countries. Unfortunately, these results are not stratified by geography, socioeconomic status, or gender. Thus, there are no data across GAVI countries to assess GAVI’s impact to reduce inequities in VPD burden within countries.

## Conclusions

This qualitative assessment is based almost exclusively on published and unpublished documents alone and relies heavily on grey literature. Steps have been taken to address reporting bias, and to focus on valid and trustworthy sources. To complement this review, future research efforts should illustrate the evolution and quantitative effects of GAVI’s efforts to address between- and within- country inequities in access to new vaccines, utilization of immunization services, access to GAVI resources, and impact on VPDs.^4^ There may also be value in complementing this work with a separate literature review of other Alliance partner document repositories (e.g. from GAVI’s donors, other multilateral partners, CSOs, etc.). Finally, there may be value in conducting an ethnographic review given the multiplicity of views across the Alliance, and the fact that others have employed these techniques effectively to chart the evolution of GAVI’s contribution to health systems strengthening [189].

Despite the limitations of this assessment, the findings potentially have great relevance for a wide array of stakeholders. By highlighting the strengths and deficiencies of GAVI’s previous strategies, policies, and programs from an equity perspective and illustrating the areas where GAVI has been successful, inconsistent, or unsuccessful in translating strategic intent into credible and robust evidence of impact, this research may be useful for those involved in the future design, implementation and evaluation of GAVI-funded immunization programs.

Within GAVI countries, GAVI’s cash-based investments are intended to bring benefit beyond immunization programs by more generally strengthening health programs. In many GAVI countries immunization programs already serve as the backbone of health supply chains. At the global level, immunization is recognized as one of the best global health ‘buys’ capable of delivering vast returns for modest investments, particularly for low- and middle-income countries [233, 234]. The results described here may be particularly useful as the Alliance embarks upon the design of the policies and plans the country programs that will define its fourth phase of operations, from 2016 through 2020, and perhaps future phases of operations thereafter. What’s more, the GAVI Alliance is a highly rated multilateral enterprise [235] embodying innovation in the global health architecture, with the GAVI Secretariat an increasingly influential actor seeking to shape the global health agenda [6]. Given this, there may be analogous lessons for other global partnerships, and particularly funding mechanisms that share characteristics with GAVI. While the governance structures, priorities and/or sectors of focus between GAVI and funding mechanisms such as the Global Fund (formerly the Global Fund to Fight AIDS, Tuberculosis, and Malaria) and the Global Partnership for Education (GPE) are different, there may be valuable lessons concerning strategy, policy and program design. All of these mechanisms should strive towards clarity on the type of equity in focus (between eligible/ineligible countries, among eligible countries, within eligible countries) and whether one of the aims for any stream of funding is to address equity of opportunity or outcomes. Furthermore, careful consideration and articulation is needed by these organizations/partnerships on whether underlying resource allocation mechanisms are built on principles of vertical or horizontal equity. Finally, this assessment points to the importance of designing programs in such a way that the equity impacts are systematically monitored, evaluated and documented. Ultimately, multi-stakeholder development funds like GAVI must ensure that strategic intent to improve equity is translated into action and demonstrable results.

In its next phase of operations, the Alliance can and likely will demonstrate that it continues to be a highly effective multi-partner enterprise, capable of learning and innovating in a world that has changed much since its inception. By building on its successes, developing more coherent and consistent approaches to address inequities in immunization between and within countries, and through monitoring and evaluating progress, GAVI will be well-positioned to bring the benefits of vaccination to previously unreached and underserved communities, ultimately contributing towards universal health coverage and sustainable development.

## Additional files

Additional file 1:
**Completed PRISMA checklist.** (DOC 67 kb) Additional file 2:
**Detailed literature results.** (XLSX 79 kb)

## Abbreviations

GAVI: Global Alliance for Vaccines and Immunization
Phase I: GAVI’s first phase of operations, 2000–2005
Phase II: GAVI’s second phase of operations, 2006–2010
Phase III: GAVI’s third phase of operations, 2011–2015
CVI: Children’s Vaccine Initiative
HepB: Hepatitis B
Hib: *Haemophilus Influenzae* type b
YF: yellow fever
NIP: National Immunization Program
GNP: Gross National Product
SNA: System of National Accounts
GNI: Gross National Income
LICs: low income countries
AD: auto-disable syringes
GOI: Government of India
WHO: World Health Organization
UNICEF: United Nations Children’s Fund
DTP3: third dose of diphtheria-tetanus-pertussis vaccines
NVS: new vaccine support
ISS: immunization services support
CLUCs: countries with large numbers of unimmunized children
LICUS: low income countries under stress
DHS: demographic and health surveys
MICS: multi-indicator cluster survey
HIV/AIDS: human immunodeficiency virus / acquired immunodeficiency syndrome
VIS: vaccine investment strategy
IFFIm: innovative financing facility for immunization
PCV: pneumococcal conjugate vaccines
RV: rotavirus vaccines
LMICs: lower-middle income countries
HPV: human papillomavirus
JE: Japanese Encephalitis
MenA: *Neisseria meningitides* group A
IPV: inactivated polio vaccine
IRC: Independent Review Committee
IDA: International Development Association
VIG: vaccine introduction grant
MNTE: maternal and neonatal tetanus elimination
HSS: health systems strengthening
CSOs: Civil Society Organizations
RED: Reach Every District
LDCs: least developed countries
PBF: performance-based funding
MICs: middle income countries
MCV1: first dose of measles-containing vaccines
MDGs: millennium development goals
GPEI: global polio eradication initiative
TA: technical assistance
VPD: vaccine preventable disease
EPI: expanded programme of immunization

## Footnotes

1 The term “GAVI countries” is generally employed to denote both GAVI eligible countries as well as countries in the process of graduating from GAVI support. The specific definitions of eligible and graduating countries are delineated in GAVI’s eligibility policy [135] and graduation procedures [236].

2 In the GAVI Alliance Evaluation Workplan 2015 report to GAVI’s Evaluation Advisory Committee, the GAVI Secretariat notes that it is currently conducting, or planning to commence *End-of-Support Evaluations* in at least two graduated countries, Albania and Bosnia & Herzegovina. The report mentions an *Advance Market Commitment (AMC) Impact Evaluation* and *Health Systems Strengthening Grant Evaluations*, both due to commence in 2015 [26].

3 One paper on this topic does exist although the discourse is at a very high level and since it was published in 2009, it does not comment on the evaluated impacts of Phase II and progress during Phase III [237]. Another piece of work was commissioned by the GAVI Secretariat for internal purposes, to provide a stock-take of how the GAVI Alliance defines, measures and seeks to address equity in Phase III. This working paper is not in the public domain and does not assess GAVI’s approach to addressing inequities has changed over time through its programs and policies, nor does it set out GAVI’s equity impacts on opportunity versus outcomes. [Vaccine Implementation Technical Assistance Consortium (VITAC), “The GAVI Alliance and equity: immunisation for all,” PATH, Geneva, 2013 Unpublished].

4 Separate to this qualitative assessment of literature, analyses of longitudinal immunization program performance data have been undertaken to assess more objectively, the possible impact of GAVI’s approaches on observed between- and within- country inequities in immunization. These analyses are described in a separate forthcoming paper.

5 In this review, the terms *inequity* and *disparity* are used interchangeably. These terms are defined here as differences that are unnecessary, avoidable, unfair and unjust across any dimension (e.g. wealth, ethnicity, geography, gender) [238, 239]. The term *inequality*, while related to inequity, is used in this paper specifically with reference to gender since unlike socioeconomic, ethnographic or other need-based differences, no amount of difference according to gender is necessary, fair or just.

6 It is acknowledged that in some large and often federated states, the roll out of new vaccines is phased over two or more years such that a NIP schedule in one region of a country may—for a time—be different than in another part of the country. It is also acknowledged that in a handful of countries that are characterized by markedly heterogeneous and biodiverse environments, risks of certain VPDs may be much more concentrated in some regions than others, and as such, vaccination schedules may differ slightly from one part of the country to another. However, these are exceptions rather than the rule, and as such are not explicitly considered in this review.

7 GAVI Committee papers usually cover the same topics as are eventually reviewed by the GAVI Board only in more detail, or in a less complete state. As such, Committee papers were only included where they provided important additional information (relevant to the assessment) than could be found in a Board paper.

8 Following GAVI’s formation, three goals were specified: (1) Increase coverage – boost the number of children whose lives can be saved with the remarkably effective tool of vaccination. (2) Shorten the time span between registration of a vaccine product in the marketplace and its full-scale use in the developing world. (3) Accelerate the development and introduction of new vaccines [42].

9 Phase I ran from 2000–2005 [17], while the Phase II strategy covered the period 2007–2010 [58]. The year 2006 was a transition year of sorts, in which the new Phase II strategy was developed.

10 Two operating principles in GAVI’s Phase III strategy that describe GAVI’s principles vis-à-vis addressing inequities: “Focusing on innovation, efficiency, equity, performance and results”; and “Ensuring gender equity in all areas of engagement” [61, 144].

11 GAVI’s target in Phase III with respect to equity improvement is as follows: To improve equity in immunisation coverage from baseline levels as measured by the performance indicator—the percentage of GAVI countries where the DTP3 coverage in the lowest wealth quintile is ±20 percentage points of the coverage in highest wealth quintile [240].

12 The strategic objective in GAVI’s Phase III strategy that articulates GAVI’s aims with respect to equity is as follows: “Increase equity in access to services” [61, 144].

13 In Phase III, GAVI renamed the Workplan as ‘the Business Plan’ – The name change signified a more rigorous approach that attempted to link back TA activities through a coherent logical framework to the overarching strategy.

14 Four of the countries identified as having large disparities in immunization coverage overlapped with countries characterized by low coverage (i.e. DTP3 < 70 % nationally). In these countries, WHO and UNICEF have co-led TA efforts to simultaneously improve both overall national coverage rates and geographic inequities in subnational coverage.

15 In order to evaluate vaccine portfolios, the VIS IRC used the following seven priorities: i). Prioritise potential integration with EPI schedule (<12 months); ii). Prioritise potential integration with an extended EPI schedule (<18 months); iii). Focus on highly effective vaccines (>75 % effectiveness in GAVI countries); iv). Focus on diseases for which no adequate treatment is currently available; v). Focus on diseases for which no adequate prevention is currently available; vi). Prioritise vaccines that address inequity of the poor (relating to diseases disproportionally affecting the poor, or to vaccines specifically beneficial to the poor); vii). Prioritise vaccines that address gender inequity (relating to diseases disproportionally affecting one sex, or to vaccines specifically beneficial to one sex).

16 While GAVI-funded MenA vaccine campaigns commenced in a handful of countries at the end of phase II (along with support for the MenA vaccine stockpile) following the approval of a MenA vaccine investment case [86, 87], the majority of these vaccines were planned for roll out in phase III as and when appropriate conjugated vaccines became available.

17 While it is not well-known, at the time of GAVI’s first three Board meetings, the list of eligible countries was initially proposed (in the Board materials) as being defined based on those countries with an annual GNP/per capita equal to or less than US $1,000 *and* a total national population of less than 150 million people [49, 107]. This ruled out three large countries: China, India and Indonesia. However, the strict population criterion was removed, and replaced with a different approach to large countries as discussed in the section entitled 'Large countries and budget cap policies' of this paper.

18 In 1999, at the time of the first Board meeting, there were initially 71 eligible countries [68 countries with per capita GNP ≤ US $1,000 and a population less than 150 million, three countries with uncertain per capita GNP but anticipated to be below or close to $1,000 (Cuba, Democratic Peoples’ Republic of Korea, and Ukraine)]. In addition, there were three further more countries with per capita GNP ≤ US $1,000 and a population above 150 million (China, India and Indonesia) whom, as noted in the footnote above and in the section of this paper entitled 'Large countries and budget cap policies' would be considered partially eligible through the remainder of phase I [116]. In 2002, following the country’s formation (and recognition by the United Nations), Timor-Leste was added to the list of eligible countries taking the total number of GAVI countries to 75 through the remainder of Phase I.

19 In 2003, the World Bank adopted the 1993 System of National Accounts (SNA) and as such changed its terminology from GNP per capita to Gross National Income (GNI) per capita. This alteration however, did not have an impact on the country statistics themselves or the ranking that informed GAVI’s country eligibility [241].

20 An update of the eligibility criteria reference data was considered midway through Phase I, but it was decided to hold the eligibility policy constant to ensure predictability [242, 243].

21 At the time, this funding was referred to as the *New and Under-used Vaccines sub-account* from the Global Fund for Children’s Vaccines (The Fund).

22 At the time, this funding was referred to as the *Immunization Services sub-account* of The Fund.

23 At the time of the update, 26 GAVI-eligible countries, representing 55 % of GAVI’s birth cohort, were above the US $1,000 GNI per capita threshold according to the latest GNI data (published by the World Bank for the financial year 2008). In addition, there were 23 countries not eligible for GAVI support whose incomes were below that of the wealthiest eligible GAVI country [145].

24 It is recognized that the terms “poor countries” and “poor people” are pejorative and no longer commonly used. However, they are used here only because it the parlance used in GAVI policy documents, Committee and Board papers during the time.

25 In June 2010, following slow progress to develop and implement a mechanism to improve coverage in countries with low coverage, the GAVI Board decided to temporarily suspend the November 2009 decision of the Board to raise the DTP3 filter to 70 % thereby re-establishing the filter threshold at 50 % for 2010 round of new vaccine support applications – and until GAVI’s new performance-based funding had been designed and launched.

26 The GOI also selected high-performing districts as it was felt these would be better placed to monitor and manage AEFIs and vaccine wastage.

27 The HSS evaluation provides the following additional information: “*The approach to equity is mixed. In some countries certain districts or provinces have been targeted based on a range of criteria, including current immunisation performance, availability of infrastructure/transport/human resources as well as socio-economic indicators: Vietnam, Bhutan, Burundi, DRC, Honduras (focused on 104 municipalities), Nicaragua (which focused on specific municipalities), Zambia (12 districts), Yemen (64 districts), Cambodia (10 ODs), Nepal (varies by component), Sri Lanka and Ghana all fall into such category. However, there is often little focus on ensuring that those most in need within those geographical areas are targeted. This is an important distinction in countries where, for instance, socio-economic status plays a higher role in accessing health care than geographical location. In other countries a national approach is adopted in some cases more because all areas are seen as equally poor (Liberia, Sierra Leone) or others where it was seen as the best way to move forward (as in Rwanda, where new districts might not have had at the time of design sufficient planning experience to enable a more differentiated district approach*)” [22].

28 The ten countries are Afghanistan, Bolivia, Democratic Republic of Congo, Ethiopia, Indonesia, Mozambique, Pakistan, Ghana, Burundi, and Georgia (although Bolivia, Georgia and Mozambique had not received CSO funding support by the time of the independent evaluation.

29 Specifically, in June 2001 the GAVI Board adopted the following definition of financial sustainability: “Although self-sufficiency is the ultimate goal, in the nearer term sustainable financing is the ability of a country to mobilize and efficiently use domestic and supplementary external resources on a reliable basis to achieve current and future target levels of immunization performance in terms of access, utilization, quality, safety and equity”. (Emphasis added) [146].

30 GAVI’s original co-financing policy country groupings used the following criteria: GNI per capita (i.e. countries with a GNI p.c. ≤ US $1,000 versus countries with a GNI p.c. >US $1,000), UN development status (i.e. least developed countries (LDCs) versus non-LDCs), and fragility (i.e. defined by the World Bank as a ‘Fragile State’) [203].

31 It is recognized that the term “fragile states” is considered pejorative and no longer commonly used. It is used here only because it reflected the parlance used in GAVI policy and strategy documents and Board papers, particularly during Phase II.

32 The Bank uses two criteria to classify countries as LICUS: (i) low-income; and (ii) poor performance on Country Policy and Institutional Assessment (CPIA). Lists of the countries classified by the World Bank as LICUS for the financial years 2006–2009 can be found online [244].

33 Other objectives of GAVI’s pilot prioritization mechanism are as follows: Maximize overall health impact (i.e. reduction of disease burden); Maximize value for money (i.e. cost-effectiveness); Reinforce the financial sustainability of immunization programs; Focus GAVI’s support on countries with the greatest need/least ability to pay; Ensure country readiness for use of GAVI-supported vaccines [226].

34 Roughly one third of GAVI countries had DTP3 coverage below 50 %, and roughly one sixth of GAVI countries had coverage below 40 % in 1999.
